# ZMYND8 Reads H3K36me2 to Activate CEBPE Transcription and Suppress Multiple Myeloma Progression through the Inhibition of Adaptive UPR Pathways

**DOI:** 10.1002/advs.202409219

**Published:** 2025-05-10

**Authors:** Jiaxuan Xu, Xiaoqing Dong, Yue Peng, Jie Yan, Peipei Xu, Feiyu Li, Suwen Zhang, Lanxin Chen, Xingjun Meng, Kangning Wang, Mengying Xing, Wenyang Li, David C. S. Huang, Quan Zhao, Bing Chen

**Affiliations:** ^1^ Department of Hematology Nanjing Drum Tower Hospital, Affiliated Hospital of Medical School The State Key Laboratory of Pharmaceutical Biotechnology China‐Australia Institute of Translational Medicine School of Life Sciences Nanjing University Department of Hematology Nanjing Drum Tower Hospital Clinical College of Nanjing University of Chinese Medicine Nanjing 210023 China; ^2^ School of Health Preservation and Rehabilitation Nanjing University of Chinese Medicine Nanjing 210023 China; ^3^ Institute of Basic Medical Sciences Chinese Academy of Medical Sciences and Peking Union Medical College Beijing 100005 China; ^4^ The Walter and Eliza Hall Institute of Medical Research Department of Medical Biology University of Melbourne Melbourne VIC 3052 Australia

**Keywords:** carfilzomib, CEBPE, H3K36me2, multiple myeloma, ZMYND8

## Abstract

Multiple myeloma (MM) pathogenesis is closely associated with aberrant epigenetic regulation and resulting modifications, such as dimethylation of lysine 36 in histone H3 (H3K36me2). However, the recognition signature of H3K36me2 and its functional role in MM remain largely unknown. Here, the zinc‐finger MYND‐type‐containing 8 (ZMYND8) is identified as a potential reader of the H3K36me2 mark that suppresses MM progression. ZMYND8 knockdown promotes the proliferation and invasion of MM cells. Combined transcriptomic and epigenomic analyses reveal that CCAAT/enhancer‐binding protein epsilon (CEBPE) is a direct downstream target of ZMYND8. CEBPE modulates adaptive unfolded protein response (UPR) pathways through the transcriptional repression of ERN1, XBP1, and ATF6 to impair cell survival. Coimmunoprecipitation and chromatin immunoprecipitation assays show that ZMYND8 activates CEBPE expression in an H3K36me2‐dependent manner and that its Pro‐Trp‐Trp‐Pro domain is required for binding H3K36me2 modules, leading to CEBPE transcription. Low ZMYND8 expression is significantly correlated with adverse clinicopathological features and poor survival outcomes in MM patients. Furthermore, ZMYND8 upregulation increases the sensitivity of MM cells to carfilzomib. Taken together, these findings demonstrate that ZMYND8 epigenetically activates CEBPE transcription and suppresses MM cell growth by inhibiting the adaptive UPR, suggesting that ZMYND8 can be a novel therapeutic target for patients with MM.

## Graphical abstract

Zinc‐finger MYND‐type‐containing 8 (ZMYND8) activates the transcription of CCAAT/enhancer‐binding protein epsilon by recognizing dimethylation of lysine 36 in histone H3 through its Pro‐Trp‐Trp‐Pro domain, thereby inhibiting adaptive unfolded protein response pathways to suppress the proliferation and invasion of multiple myeloma (MM) cells. Upregulating the expression level of ZMYND8 enhances the efficacy of carfilzomib in the treatment of MM.

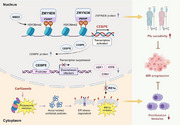

## 1 Introduction

Multiple myeloma (MM) is characterized by the abnormal proliferation of clonal plasma cells in the bone marrow.^[^ ^1^ ^]^ Among hematologic malignancies, MM ranks second in terms of morbidity and mortality, after non‐Hodgkin's lymphoma.^[^ ^2^ ^]^ Patients with MM often experience symptoms such as hypercalcemia, renal failure, anemia, and bone lesions.^[^ ^3^ ^]^ Although mainstream regimens based on proteasome inhibitors (PIs) have significantly improved patient outcomes in recent years,^[^ ^4^ ^]^ owing to the intricate biological properties of MM, patients ultimately face substantial risks of drug resistance, tumor progression, and disease relapse. Therefore, it is imperative to elucidate the underlying pathogenesis of MM and develop novel treatment strategies.

Aberrant histone H3 lysine 36 dimethylation (H3K36me2) is closely associated with chromosomal t(4;14) translocation in MM. T(4;14) translocation occurs in ≈15–20% of patients with MM, is correlated with poor prognosis, and leads to the overexpression of the histone methyltransferase nuclear receptor SET domain‐containing protein 2 (NSD2; also known as MMSET).^[^ ^5^, ^6^ ^]^ H3K36me2 is a crucial chromatin modification catalyzed by NSD2 and its dysregulation induces epigenome reprogramming in myeloma, serving as a high‐risk driver of carcinogenesis.^[^ ^7^, ^8^ ^]^ NSD2 initiates oncogenic programming and promotes myelomagenesis in an H3K36me2‐dependent manner, irrespective of the presence of t(4;14) in MM cells.^[^ ^9^ ^]^ Therefore, H3K36me2 modification is essential for myeloma initiation and progression. Epigenetically, an important link in histone modification is the recruitment of reader proteins, which mediate gene transcription and DNA repair after recognizing histone lysine modifications, and transduce molecular signals within chromatins into downstream biological effects.^[^ ^10^, ^11^ ^]^ Here, H3K36me2 modification is theoretically recognized by reader proteins containing special domains, and is dependent on the readers to produce crucial biological functions such as chromatin remodeling and gene transcription regulation.^[^ ^12^, ^13^ ^]^ However, the mechanisms by which specific reader proteins recognize H3K36me2 and mediate its downstream effects in MM are poorly understood.

Zinc‐finger MYND‐type‐containing 8 (ZMYND8) is a histone H3‐interacting protein that harbors a conserved binding module with high affinity for chromatin.^[^ ^14^ ^]^ ZMYND8 is a potential reader protein for H3K36me2 modification based on its structural domains: Pro‐Trp‐Trp‐Pro (PWWP) domain, bromodomain (BRD), and plant homeodomain (PHD), which jointly form a PHD–BRD–PWWP (PBP) structure capable of reading multiple histone methylation and acetylation marks.^[^ ^15^ ^]^ ZMYND8 has been reported to play pivotal roles in the DNA damage response, cellular growth, neural differentiation, and tumor development.^[^ ^14^, ^16^ ^]^ ZMYND8 has tumor‐suppressive effects on breast, prostate, and nasopharyngeal cancers via multiple mechanisms, such as inducing cell differentiation; inhibiting cell proliferation, invasion, and metastasis; arresting cell cycle progression; and maintaining the epithelial phenotype.^[^ ^17^, ^18^, ^19^, ^20^ ^]^ By contrast, ZMYND8 has tumor‐promoting effects on bladder cancer, colorectal cancer, and hepatocellular carcinoma through the promotion of angiogenesis, proliferation, invasion, and metastasis, as well as metabolic alterations in cancer cells.^[^ ^21^, ^22^, ^23^, ^24^ ^]^ In hematological cancers such as acute myeloid leukemia (AML), perturbing the epigenetic reader function of ZMYND8 can inhibit the AML‐dependent IRF8 transcription axis.^[^ ^25^ ^]^ Despite these findings, the biological functions and underlying mechanisms of ZMYND8 in MM still remain unclear.

In this study, we aimed to explore the biological role of ZMYND8 and the molecular mechanism involved in its recognition of H3K36me2 in MM. We first evaluated the expression level and clinical significance of ZMYND8 in patients with MM and found that low ZMYND8 expression could predict adverse clinical outcomes. Next, we studied the specific function of ZMYND8 in MM and identified CCAAT/enhancer‐binding protein epsilon (CEBPE) as a key target of ZMYND8 via high‐throughput sequencing. Cleavage under targets and tagmentation (CUT&Tag), coimmunoprecipitation (Co‐IP), and chromatin immunoprecipitation (ChIP) assays were used to determine the binding ability of ZMYND8 to H3K36me2 and its regulatory effects on CEBPE transcription. Furthermore, we explored the impact of ZMYND8 expression on the sensitivity of MM cells to PIs and found that ZMYND8 overexpression significantly enhanced the antimyeloma effect of carfilzomib (CFZ), which is expected to support the development of novel clinical therapies. This study provides a comprehensive and innovative perspective on biomarker applications, pathogenic mechanisms, and therapeutic strategies involving ZMYND8 in patients with MM.

## 2 Results

### 2.1 ZMYND8 Is Expressed at Low Levels in MM, and Its Downregulation Predicts Poor Prognosis

**Figure 1:**
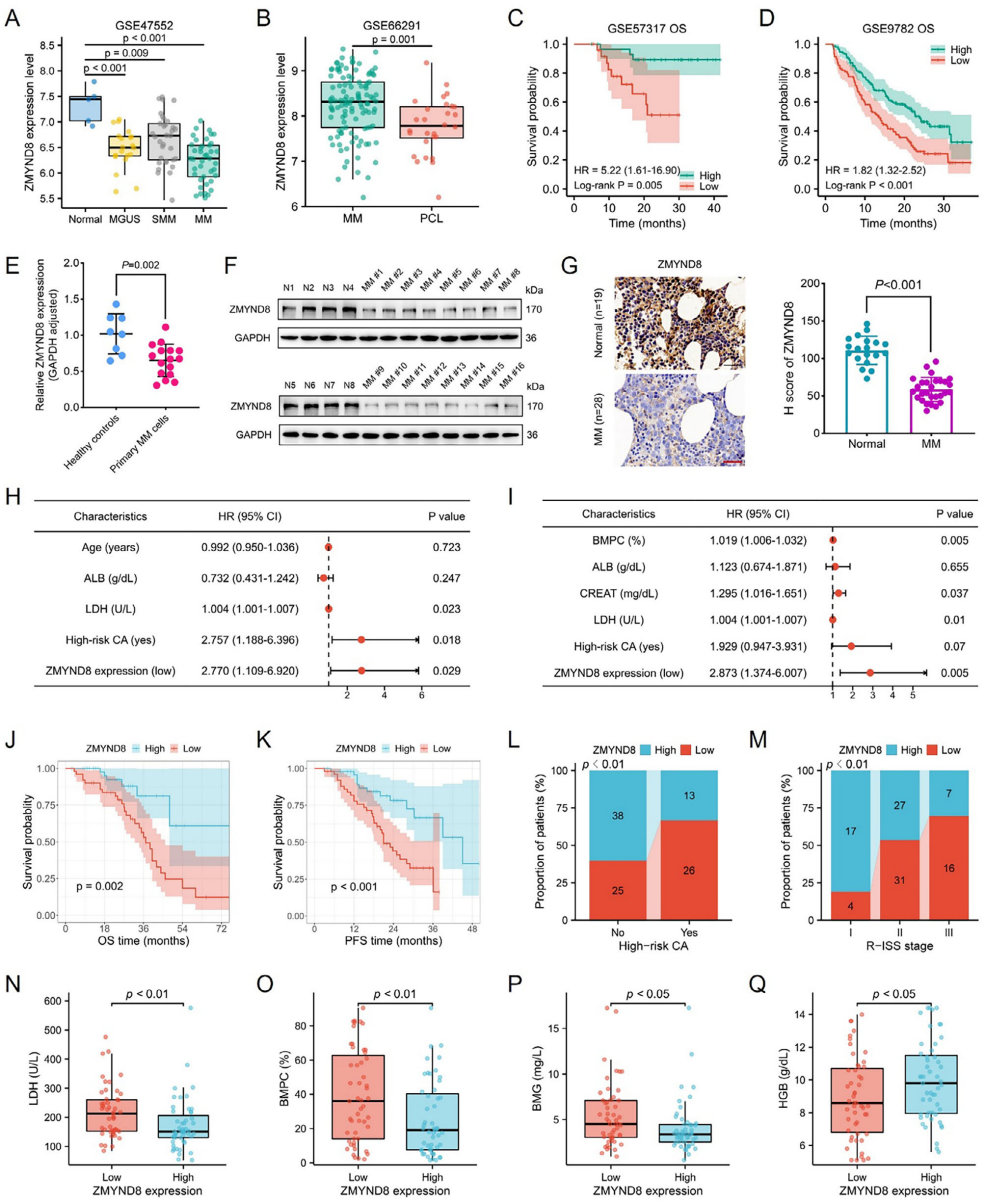
ZMYND8 downregulation is associated with unfavorable clinical features and survival outcomes in patients with multiple myeloma (MM). A) Expression levels of ZMYND8 in normal control (*n* = 5), MGUS (*n* = 20), SMM (*n* = 33), and MM (*n* = 41) samples in the GSE47552 dataset. B) Expression values of ZMYND8 in patients with MM (*n* = 114) and PCL (*n* = 28) from the GSE66291 dataset. Data in (A) and (B) are visualized with boxplots and presented as median ± interquartile range (IQR). C,D) Kaplan–Meier curves of OS for patients with high and low expression of ZMYND8 in the GSE57317 (*n* = 55) and GSE9782 (*n* = 264) datasets. E) Relative ZMYND8 mRNA expression between MM bone marrow CD138+ cells (*n* = 16) and normal controls (*n* = 8) was determined via qPCR analysis. F) Immunoblots showing the differences in ZMYND8 protein levels between healthy donors and patients with MM. G) Representative images of IHC staining (left panel) and *H*‐score quantitative analysis (right panel) of the ZMYDN8 protein in tissue samples from healthy individuals (*n* = 19) and patients with MM (*n* = 28). The ruler length is 50 µm. Data in (E) and (G) are presented as mean ± standard deviation (SD) and analyzed by Student's *t*‐test. H,I) Forest plots of multivariate Cox analysis for risk factors associated with OS (H) and PFS (I) in the MM cohort (*n* = 102). J,K) Kaplan–Meier survival curves of OS (J) and PFS (K) for patients with high and low ZMYND8 expression in the MM cohort (*n* = 102). L,M) Proportions of patients with high and low expression of ZMYND8 in different subgroups according to high‐risk CA (L) and R‐ISS stages (M). Data in (L) and (M) were compared by Chi‐squared test. N–Q) Comparison of LDH, BMPC, BMG, and HGB levels between patients with MM in the ZMYND8^high^ (*n* = 51) and ZMYND8^low^ (*n* = 51) expression groups. Data in (N–Q) are presented as median ± IQR and analyzed by Wilcoxon rank‐sum test. Abbreviations: ALB, albumin; BMG, β2‐microglobulin; BMPC, bone marrow plasma cell; CA, cytogenetic abnormality; CREAT, creatinine; HGB, hemoglobin; IHC, immunohistochemistry; LDH, lactate dehydrogenase; MGUS, monoclonal gammopathy of undetermined significance; OS, overall survival; PCL, plasma cell leukemia; PFS, progression‐free survival; R‐ISS, Revised‐International Staging System; SMM, smoldering multiple myeloma.

Analysis of the Cancer Cell Line Encyclopedia database revealed that, across 30 types of cancers, the ZMYND8 expression level was almost the lowest in MM cell lines (Figure S1A, Supporting Information). Comparison of ZMYND8 mRNA expression from normal, MM, and other myeloma stages revealed that ZMYND8 expression gradually decreased during myeloma progression (**Figure** **1**A and Figure S1B (Supporting Information)). ZMYND8 expression in plasma cell leukemia (PCL), which is a highly aggressive terminal stage of MM, was lower than that in MM and normal samples (Figure 1B and Figure S1C,D (Supporting Information)). In MM‐paired samples, ZMYND8 expression was lower at the time of relapse than at baseline (Figure S1E, Supporting Information). In particular, ZMYND8 expression was notably lower in MM samples than in monoclonal gammopathy of undetermined significance (MGUS) samples (Figure S1F, Supporting Information), and the ROC curve indicated that ZMYND8 is a robust diagnostic biomarker for discriminating between MGUS and MM (Figure S1G (Supporting Information), AUC = 0.872). Patients with low ZMYND8 expression had significantly worse overall survival (OS) than those with high ZMYND8 expression in all four MM datasets (Figure 1C,D and Figure S2A (Supporting Information)). Low ZMYND8 expression was also associated with poor event‐free survival and progression‐free survival (PFS) in the MM datasets (Figure S2B, Supporting Information). These observations were verified via western blotting and quantitative polymerase chain reaction (qPCR) analyses of clinical samples, which revealed significantly lower expression of ZMYND8 in primary MM cells than in healthy donor cells (Figure 1E,F). Immunohistochemistry (IHC) staining and quantitative analysis further confirmed the decreased expression of ZMYND8 in MM samples compared with normal controls (Figure 1G).

We collected the clinical data of patients diagnosed with MM at Nanjing Drum Tower Hospital and categorized them into two groups on the basis of the median ZMYND8 protein level detected via IHC staining. A total of 102 patients were included in the MM cohort; their baseline characteristics are shown in Table S1 (Supporting Information). There were significant differences in age, percentage of bone marrow plasma cells (BMPCs), β2‐microglobulin level, hemoglobin level, lactate dehydrogenase (LDH) level, proportion of high‐risk cytogenetic abnormalities, ISS stage, and R‐ISS stage between the high‐ and low‐ZMYND8 expression groups. Univariate and multivariate Cox analyses were performed to identify the significant prognostic factors for OS (Table S2, Supporting Information) and PFS (Table S3, Supporting Information). LDH, high‐risk cytogenetic abnormalities, and ZMYND8 expression were identified as independent prognostic indicators of OS (Figure 1H), whereas the percentage of BMPCs, creatinine level, LDH level, and ZMYND8 expression independently predicted PFS in patients with MM (Figure 1I). The Kaplan–Meier curves revealed that both the OS and PFS of patients with high ZMYND8 expression were better than those of patients with low ZMYND8 expression (Figure 1J,K). The proportion of patients with low ZMYND8 expression was prominently greater among those aged 60 years and older and those with high‐risk cytogenetic abnormalities, a high ISS stage, and an advanced R‐ISS stage (Figure 1L,M and Figure S2C,D (Supporting Information)). Additionally, LDH, BMPC infiltration, and β2‐microglobulin levels were significantly greater in patients in the low ZMYND8 expression group than in those in the high ZMYND8 expression group, while their hemoglobin levels were markedly downregulated (Figure 1N–Q). These results suggest that ZMYND8 expression is strongly associated with key clinicopathological features and can potentially be used to assess disease burden and predict survival outcomes in patients with MM.

### 2.2 ZMYND8 Suppresses Cell Proliferation and Invasion and Induces Cell Apoptosis in MM

**Figure 2:**
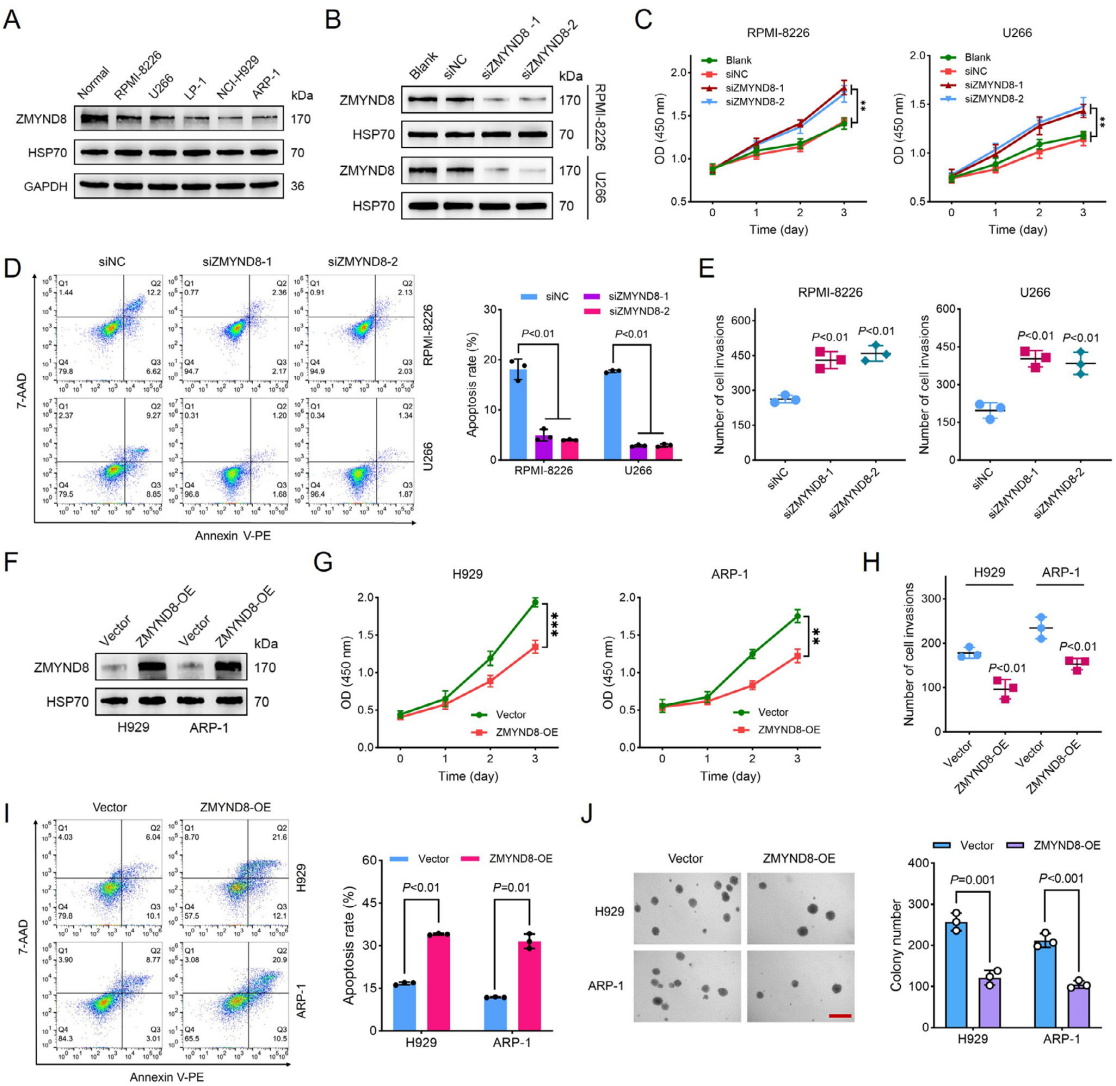
ZMYND8 inhibits the growth of MM cells in vitro. A) Immunoblots showing the ZMYND8 protein levels in five MM cell lines (RPMI‐8226, U266, LP‐1, NCI‐H929, and ARP‐1) compared with those in normal bone marrow plasma cells. The HSP70 protein was used as a reference control. B) Western blot analysis of ZMYND8 expression in RPMI‐8226 and U266 cells transfected with the negative control (siNC) or siZMYND8. C) Cell proliferation assays via CCK‐8 after ZMYND8 was silenced in RPMI‐8226 (left) and U266 (right) cells. Data in (C) are presented as mean ± SD, ** *p* < 0.01 by two‐way analysis of variance (ANOVA) test. D) Cell apoptosis was measured via flow cytometry in RPMI‐8226 and U266 cells transfected with siNC or siZMYND8 under tunicamycin‐induced stress. E) Effects of ZMYND8 silencing on the invasiveness of RPMI‐8226 (left) and U266 (right) cells, as assessed via Transwell assays. Data in (D) and (E) are presented as mean ± SD and analyzed by one‐way ANOVA test. F) The protein levels of ZMYND8 in H929 and ARP‐1 cells in the vector and ZMYND8‐overexpressing (OE) groups were analyzed via western blotting. G) CCK‐8 assays showing the effects of ZMYND8 overexpression on the proliferation of H929 (left) and ARP‐1 (right) cells. Data in (G) are presented as mean ± SD, ** *p* < 0.01, *** *p* < 0.001 by two‐way ANOVA test. H) Transwell invasion assays after ZMYND8 overexpression in H929 and ARP‐1 cells. I) Analysis of H929 and ARP‐1 cell apoptosis in the vector control and ZMYND8‐OE groups under tunicamycin‐induced stress conditions. J) Effects of ZMYND8 overexpression on the clonogenic abilities of H929 and ARP‐1 cells. Representative images of colony formation (left panel) and quantitative analysis of colony numbers (right panel) are shown for the indicated MM cells. The scale bar represents 300 µm. Data in (H–J) are presented as mean ± SD and analyzed by Student's *t*‐test.

To determine the role of ZMYND8 in the progression of myeloma, we investigated its effects on cell proliferation, invasion, and apoptosis in vitro. ZMYND8 protein levels were lower in all five MM cell lines than those in the normal BMPCs. Among the MM cell lines, ZMYND8 expression was low in H929 and ARP‐1 cells but high in RPMI‐8226 and U266 cells (**Figure** **2**A). Therefore, H929 and ARP‐1 cells were selected for ZMYND8 overexpression, and RPMI‐8226 and U266 cells were used for ZMYND8 silencing. Through small interfering RNA (siRNA) transfection, ZMYND8 was knocked down in the RPMI‐8226 and U266 cell lines, as confirmed by qPCR and western blot analyses (Figure 2B and Figure S3A (Supporting Information)). CCK‐8 assays revealed that ZMYND8 silencing increased the proliferative ability of both MM cell lines (Figure 2C). Compared with those in the negative control groups, the apoptosis rates in the siZMYND8 group were markedly lower (Figure 2D). Moreover, ZMYND8 silencing significantly promoted the invasion of RPMI‐8226 and U266 cells compared with the negative controls (Figure 2E).

Stable ZMYND8‐overexpressing (OE) H929 and ARP‐1 cell lines were constructed, and ZMYND8 overexpression was validated via qPCR and western blotting (Figure 2F and Figure S3B (Supporting Information)). CCK‐8 and Transwell assays revealed that ZMYND8 overexpression significantly inhibited the proliferation and invasion of MM cells (Figure 2G,H). Compared with that in the vector control cells, the degree of apoptosis in ZMYND8‐OE cells was slightly greater (Figure S3C, Supporting Information), and ZMYND8 overexpression significantly promoted tunicamycin‐induced apoptosis under stress conditions (Figure 2I). Similarly, ZMYND8 upregulation effectively weakened the colony‐forming ability of H929 and ARP‐1 cells (Figure 2J). These findings indicate that ZMYND8 markedly inhibits the proliferation and invasion of MM cells.

### 2.3 CEBPE Is a Direct Target of ZMYND8 and Functions as a Tumor Suppressor in MM

**Figure 3:**
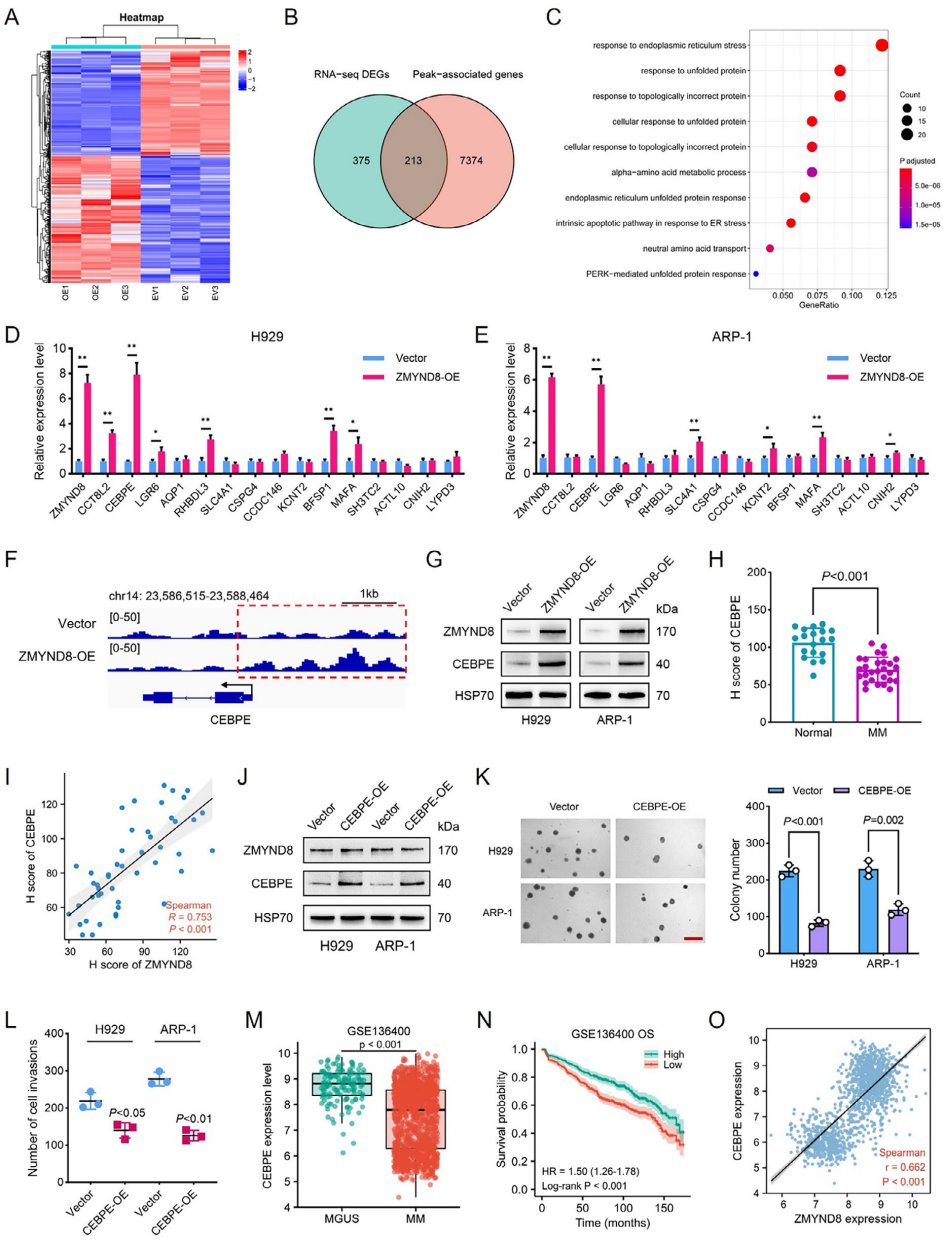
CEBPE was identified as a direct downstream target of ZMYND8. A) Heatmap depicting differential gene expression after stable ZMYND8 overexpression in H929 cells. Red indicates relatively highly expressed genes, and blue represents relatively low gene expression. RNA sequencing (RNA‐seq) was performed on three independent replicate samples from the empty vector (EV) group and the ZMYND8‐OE group. B) Venn diagram showing the intersection of differentially expressed genes (DEGs) identified via RNA‐seq and ZMYND8 peak‐associated genes identified via cleavage under targets and tagmentation (CUT&Tag). C) Gene Ontology (GO) analysis of overlapping genes between the RNA‐seq DEGs and peak‐associated genes. D,E) Relative mRNA expression levels of the top 15 overlapping genes verified via qPCR after ZMYND8 overexpression in H929 (D) and ARP‐1 (E) cells. GAPDH was used as an internal reference gene. Data in (D) and (E) are presented as mean ± SD, * *p* < 0.05, ** *p* < 0.01 by Student's *t*‐test. F) CUT&Tag profiling tracks of ZMYND8 at the CEBPE gene locus in the ZMYND8‐OE group compared with the vector control group. G) Protein levels of CEBPE in H929 and ARP‐1 cells in the vector and ZMYND8‐OE groups determined via western blot analysis. H) *H*‐score analysis of the CEBPE protein in bone marrow samples from healthy individuals (*n* = 19) and patients with MM (*n* = 28). Data in (H) are presented as mean ± SD and analyzed by Student's *t*‐test. I) Spearman correlation analysis of the protein levels of ZMYND8 and CEBPE in clinical immunohistochemical samples (*n* = 47). J) Protein levels of ZMYND8 and CEBPE in the vector and CEBPE‐OE groups of H929 and ARP‐1 cells. K) Methylcellulose colony formation assays and quantitative analysis of clone numbers after CEBPE overexpression in MM cells. The scale bar represents 300 µm. L) Cell invasion assays after CEBPE overexpression in H929 and ARP‐1 cells. Data in (K) and (L) are presented as mean ± SD and analyzed by Student's *t*‐test. M) Comparison of CEBPE expression levels between patients with MGUS (*n* = 131) and MM (*n* = 1293). Data in (M) are presented as median ± IQR and analyzed by Wilcoxon rank‐sum test. N) Kaplan–Meier curves of overall survival (OS) for patients with high and low expression of CEBPE in the GSE136400 dataset (*n* = 1293). O) Spearman correlations of ZMYND8 expression with CEBPE expression in patients with MM in the GSE136400 dataset (*n* = 1293).

To investigate the gene expression profile regulated by ZMYND8, transcriptome sequencing was performed in H929 cells stably transfected with the control vector or ZMYND8‐OE plasmid. In total, 588 differentially expressed genes (DEGs) were identified, including 323 upregulated and 265 downregulated genes (**Figure** **3**A). Gene Ontology (GO) analysis revealed that these genes were associated with cell differentiation, endoplasmic reticulum (ER) stress pathways, and extracellular vesicles (Figure S4A, Supporting Information). KEGG enrichment analysis revealed that the DEGs were involved in cancer‐related pathways, such as the PI3K‐AKT, JAK‐STAT, and MAPK signaling pathways (Figure S4B, Supporting Information). CUT&Tag assays were subsequently performed to assess the chromatin‐binding status of ZMYND8 in H929 cells. We identified 7587 genes with significant ZMYND8 peaks and observed that 213 peak‐associated genes overlapped with the DEGs identified via RNA sequencing (RNA‐seq) (Figure 3B). GO analysis revealed that these overlapping genes were enriched mainly in the response to ER stress and unfolded proteins (Figure 3C). Next, we conducted qPCR validation of the 15 genes with the greatest changes in expression among these overlapping genes and found that CEBPE upregulation was the most pronounced in both H929 and ARP‐1 cells (Figure 3D,E). CEBPZ was also upregulated in ZMYND8‐OE cells, whereas CEBPA, CEBPB, CEBPD, and CEBPG levels were negatively correlated with ZMYND8 expression (Figure S4C, Supporting Information). CUT&Tag analysis revealed that ZMYND8 overexpression resulted in a greater enrichment of ZMYND8 in the CEBPE promoter region compared with the vector control (Figure 3F). ZMYND8 overexpression also significantly increased CEBPE protein expression in H929 and ARP‐1 cells (Figure 3G). These observations suggest that CEBPE is a crucial target that is transcriptionally regulated by ZMYND8.

IHC analysis revealed that CEBPE was downregulated in patients with MM compared with healthy donors (Figure 3H and Figure S4D (Supporting Information)), and there was a strong positive relationship between ZMYND8 and CEBPE protein expression (Figure 3I, *r* = 0.753). We constructed two stable CEBPE‐OE cell lines and confirmed their overexpression efficiency via western blotting and qPCR (Figure 3J and Figure S4E (Supporting Information)). Notably, CEBPE overexpression did not affect ZMYND8 mRNA or protein expression. CEBPE upregulation significantly attenuated the proliferative capacity of H929 and ARP‐1 cells (Figure S4F, Supporting Information). Compared with that in the vector control group, stable overexpression of CEBPE markedly reduced the number of colonies formed in the CEBPE‐OE group (Figure 3K). Additionally, the invasive capacity of CEBPE‐OE cells was lower than that of the vector controls (Figure 3L). In the GSE13400 dataset, CEBPE was expressed at lower levels in patients with MM than in those with MGUS (Figure 3M). The ROC curve suggested that CEBPE expression could be used to distinguish MGUS from MM (Figure S4G (Supporting Information), AUC = 0.793). Low CEBPE expression was significantly associated with poorer OS and PFS in patients with MM (Figure 3N and Figure S4H (Supporting Information)). Furthermore, CEBPE expression was positively correlated with ZMYND8 expression in MM samples from the GSE136400 dataset (Figure 3O, *r* = 0.662). Taken together, these findings indicate that CEBPE acts as a tumor suppressor and inhibits the proliferation and invasion of MM cells.

### 2.4 Restoration of CEBPE Rescues the Prosurvival Effect Induced by ZMYND8 Knockdown

**Figure 4:**
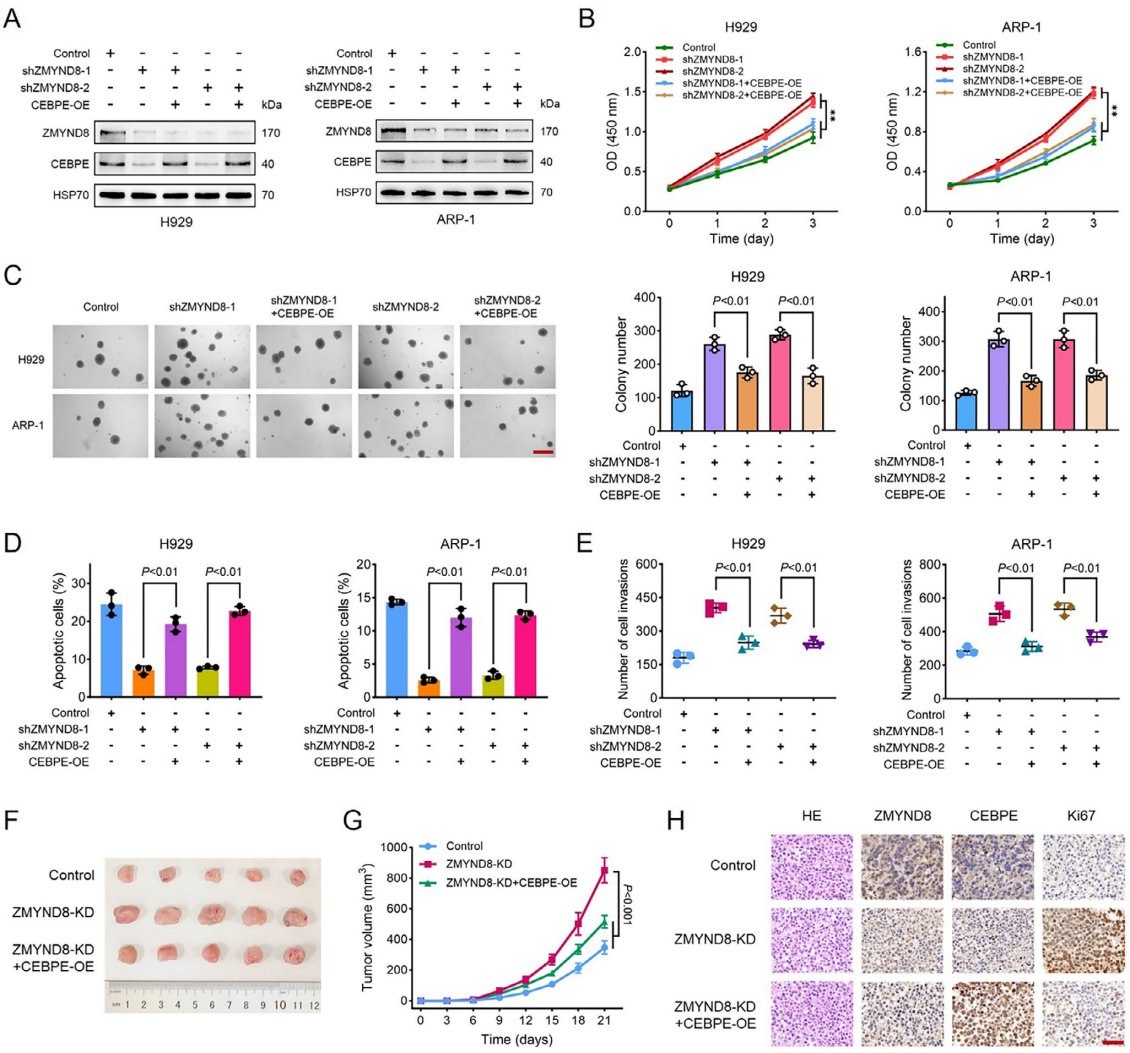
ZMYND8 knockdown promotes MM cell survival in vitro and in vivo by regulating CEBPE. A) Protein expression levels of ZMYND8 and CEBPE in H929 and ARP‐1 cells stably expressing scramble control or ZMYND8 short hairpin RNA (shRNA) plasmids. B,C) Cell proliferation assays via CCK‐8 (B) and methylcellulose colony formation (C) after ZMYND8 knockdown (KD) or ZMYND8‐KD + CEBPE‐OE in H929 and ARP‐1 cells. The scale bar represents 300 µm. Data in (B) are presented as mean ± SD, ** *p* < 0.01 by two‐way ANOVA test. D) Cell apoptosis in response to tunicamycin treatment was measured via flow cytometry in the negative control, shZMYND8, and shZMYND8 + CEBPE‐OE groups. E) Number of invading cells in the negative control, shZMYND8, and shZMYND8 + CEBPE‐OE groups as determined via Transwell assays. Data in (C–E) are presented as mean ± SD and analyzed by one‐way ANOVA test. F,G) Images of tumor size (F) and tumor volume (G) in the control, ZMYND8‐KD, and ZMYND8‐KD + CEBPE‐OE groups. Data in (G) are presented as mean ± SD and analyzed by two‐way ANOVA test (*n* = 5). H) Hematoxylin and eosin (HE) and immunohistochemical analysis of the indicated proteins in tumor xenografts excised from the mice in each experimental group. The ruler length is 50 µm.

We subsequently investigated whether restoring CEBPE expression could reverse the phenotypic changes in MM cells induced by ZMYND8 knockdown (KD). Two specific short hairpin RNAs (shRNAs) were used to establish stable ZMYND8‐KD MM cell lines. Western blot analysis revealed that ZMYND8 knockdown resulted in the downregulation of CEBPE expression, and exogenous overexpression of CEBPE was validated in ZMYND‐KD cells (**Figure** **4**A). CCK‐8 and colony formation assays indicated that ZMYND8 downregulation significantly promoted cell proliferation, whereas CEBPE overexpression strongly counteracted the proliferation‐promoting effects induced by ZMYND8 silencing (Figure 4B,C). Increased CEBPE expression rescued the antiapoptotic and proinvasive effects of ZMYND8 downregulation in H929 and ARP‐1 cells (Figure 4D,E). Next, we performed xenograft experiments to verify the CEBPE‐mediated inhibitory effects on tumor growth. Consistent with the in vitro findings, ZMYND8 knockdown markedly promoted MM growth in tumor‐bearing mice, whereas CEBPE overexpression effectively reversed the tumor‐promoting effects mediated by the loss of ZMYND8 (Figure 4F,G and Figure S5A (Supporting Information)). IHC analysis of tumor tissues revealed that the protein levels of ZMYND8 and CEBPE in the ZMYND8‐KD group were significantly lower than those in the control group and that the Ki67 levels increased after ZMYND8 knockdown. However, Ki67 expression significantly decreased with the upregulation of CEBPE, suggesting an attenuated tumor proliferation capacity (Figure 4H and Figure S5B–D (Supporting Information)). Collectively, these results indicate that ZMYND8 suppresses MM cell growth by regulating CEBPE expression both in vitro and in vivo.

### 2.5 CEBPE Regulates ER Stress by Directly Repressing ERN1, XBP1, and ATF6 Expression

**Figure 5:**
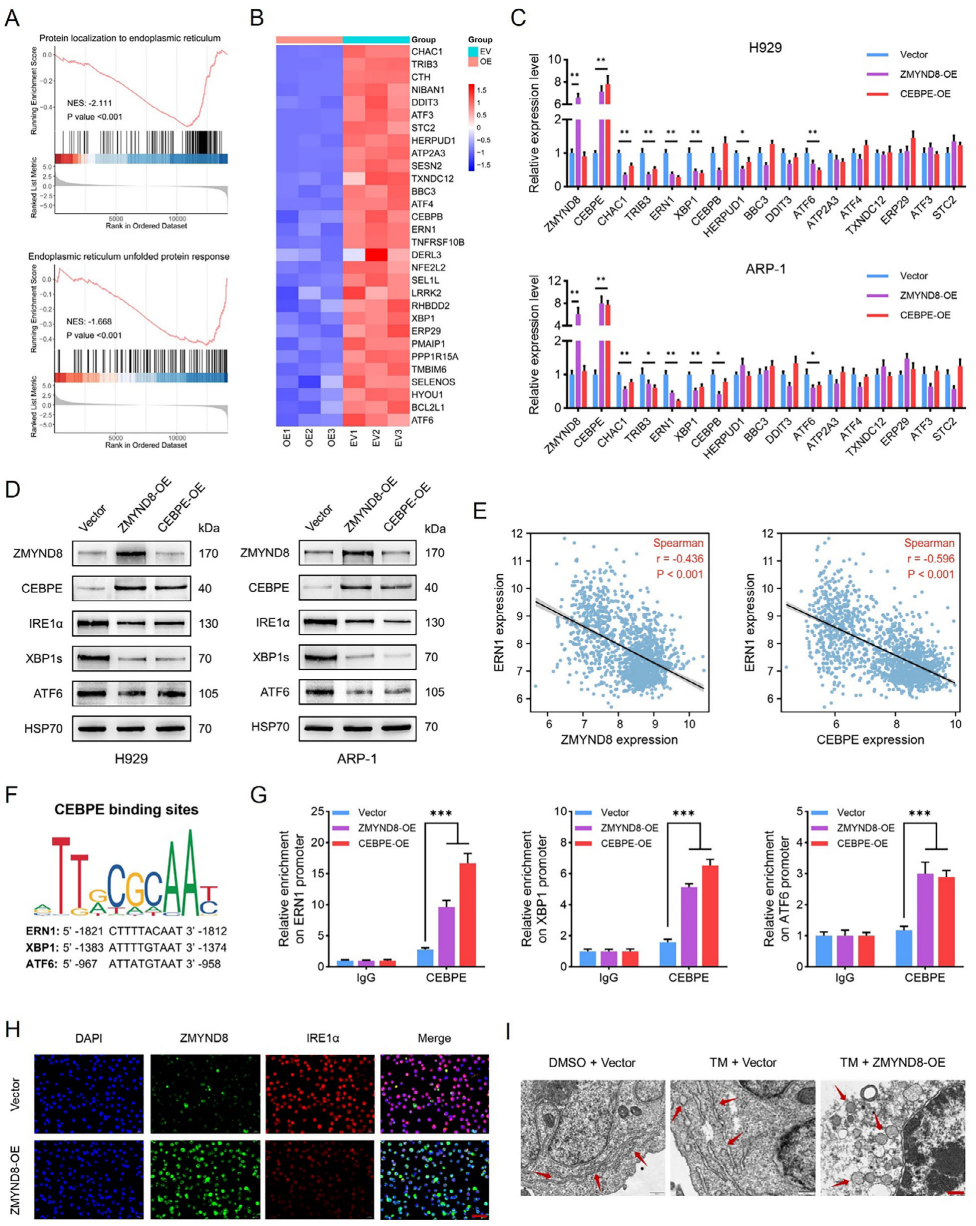
CEBPE regulates the transcription of endoplasmic‐reticulum (ER) stress‐related genes. A) Gene set enrichment analysis (GSEA) of proteins localized to the ER (upper panel) and ER unfolded protein response (lower panel) pathways in H929 cells following ZMYND8 overexpression. NES, normalized enrichment score. B) Heatmap of the RNA sequencing data showing the top 30 DEGs related to ER stress in the vector control and ZMYND8‐OE groups. C) Relative mRNA levels of a panel of key ER‐stress‐related genes validated via qPCR analysis in H929 (upper) and ARP‐1 (lower) cells transfected with the vector control, ZMYND8‐OE, or CEBPE‐OE plasmid. GAPDH was used as an internal reference. Data in (C) are presented as mean ± SD, * *p* < 0.05, ** *p* < 0.01 by one‐way ANOVA test. D) Western blot analysis of the indicated proteins in H929 and ARP‐1 cells transfected with the vector control, ZMYND8‐OE, or CEBPE‐OE plasmid. E) Spearman correlations of ERN1 expression with ZMYND8 (left) and CEBPE (right) expression in MM samples in the GSE136400 dataset (*n* = 1293). F) Motif analysis of CEBPE binding sites and possible binding regions of CEBPE in the promoters of ERN1, XBP1, and ATF6, as predicted via the JASPAR database (https://jaspar.genereg.net). G) Chromatin immunoprecipitation (ChIP)–qPCR analysis of relative CEBPE enrichment within the promoter regions of ERN1 (left), XBP1 (middle), and ATF6 (right) in H929 cells transfected with the vector control, ZMYND8‐OE, or CEBPE‐OE plasmid. The fold enrichment was calculated relative to that of the IgG control. Data in (G) are presented as mean ± SD, *** *p* < 0.001 by two‐way ANOVA test. H) Immunofluorescence was used for the detection of ZMYND8 and IRE1α in H929 cells transfected with the vector control or ZMYND8‐OE plasmid. The scale bar represents 50 µm. I) Transmission electron microscopy (TEM) was used to observe the ultrastructure of endoplasmic reticulum of H929 cells in the dimethyl sulfoxide (DMSO) + vector, tunicamycin (TM) + vector, and TM + ZMYND8‐OE groups. Red arrows indicate representative endoplasmic reticulum. The scale bar represents 0.5 µm.

To elucidate the specific mechanism by which CEBPE hinders MM cell growth, we hypothesized that this effect may be related to ER stress pathways considering previous enrichment analysis results. Gene set enrichment analysis (GSEA) revealed that the “protein localization to ER” and “ER unfolded protein response (UPR)” pathways were significantly inhibited in the ZMYND8‐OE group (**Figure** **5**A). The top‐ranked DEGs associated with ER stress were downregulated after ZMYND8 overexpression, as shown in the heatmap in Figure 5B. We examined the mRNA expression of key ER‐stress‐associated genes by qPCR analysis in H929 and ARP‐1 cells. Among these candidates, the levels of ATF6, CHAC1, ERN1, TRIB3, and XBP1 were significantly decreased in both the ZMYND8‐OE and CEBPE‐OE groups in both MM cell lines (Figure 5C). Given their roles in ER stress pathways, we selected the key effectors ERN1, XBP1, and ATF6 in the adaptive UPR for further investigation. Compared with those in the vector controls, the protein levels of IRE1α (encoded by ERN1), XBP1s, and ATF6 were also markedly reduced upon ZMYND8 or CEBPE overexpression in MM cells (Figure 5D). Notably, ERN1 mRNA expression was inversely correlated with ZMYND8 or CEBPE expression in MM samples (Figure 5E).

We used the JASPAR database to predict the most significant CEBPE binding sites in the proximal promoter regions of ERN1, XBP1, and ATF6 (Figure 5F). ChIP analysis revealed a significant increase in CEBPE enrichment at the promoters of ERN1, XBP1, and ATF6 following the overexpression of either ZMYND8 or CEBPE in both H929 and ARP‐1 cells (Figure 5G and Figure S6A (Supporting Information)). Among these three genes, the increase in CEBPE at the ERN1 promoter was the most pronounced, followed by that at the promoters of XBP1 and ATF6. Furthermore, CUT&Tag data revealed that alterations in ZMYND8 expression did not significantly affect ZMYND8 enrichment at the ERN1 and ATF6 loci and induced only a slight change in ZMYND8 enrichment at the XBP1 promoter (Figure S6B, Supporting Information). ChIP–qPCR analysis revealed that the enrichment of ZMYND8 in the promoter regions of these three genes was low in normal H929 cells, and there was no significant change in ZMYND8 enrichment even after ZMYND8 or CEBPE was overexpressed (Figure S6C, Supporting Information). Moreover, immunofluorescent findings confirmed that ZMYND8 overexpression significantly inhibited the expression of IRE1α in MM cells (Figure 5H). The results of transmission electron microscopy (TEM) indicated that under tunicamycin treatment, ZMYND8 overexpression caused significant swelling and vesiculation of the endoplasmic reticulum in MM cells (Figure 5I). These data indicate that ZMYND8 does not directly regulate the expression of ERN1, XBP1, or ATF6 but indirectly suppresses prosurvival ER stress pathways through CEBPE‐mediated transcriptional inhibition of these ER‐stress‐related effectors.

### 2.6 ZMYND8 Activates CEBPE Expression by Interacting with H3K36me2 via Its PWWP Domain

**Figure 6:**
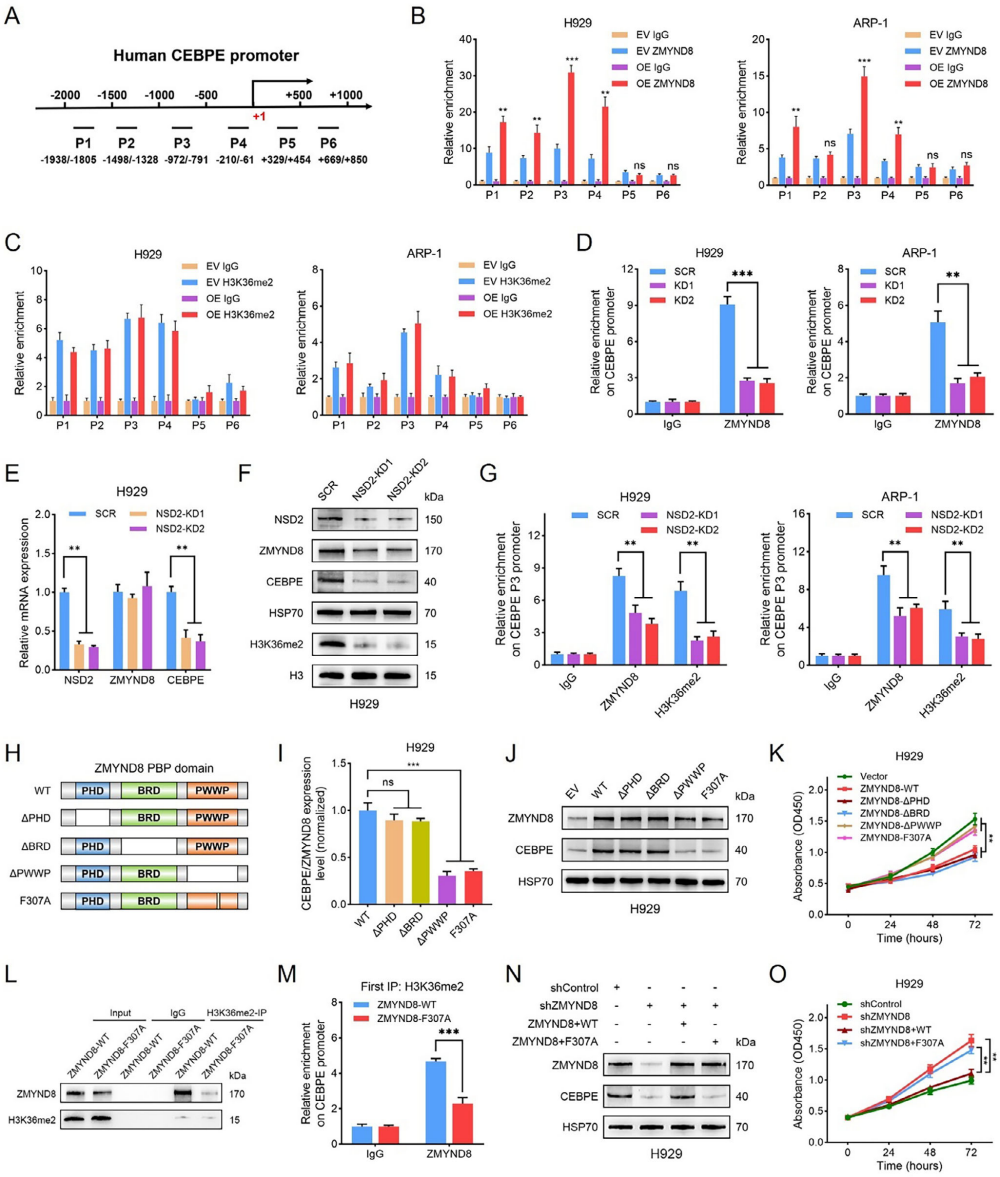
ZMYND8 recognizes H3K36me2 through its PWWP domain to modulate CEBPE transcription. A) Schematic illustration of the human CEBPE gene promoter used for ChIP analysis. The transcriptional start site (TSS) was designated the “+1” position, and the positions of the designed ChIP–qPCR primers were labeled relative to the TSS. B,C) ChIP–qPCR analysis of ZMYND8 (B) and H3K36me2 (C) enrichment on the CEBPE promoter in the vector control and ZMYND8‐OE groups of H929 and ARP‐1 cells. D) ChIP–qPCR analysis of ZMYND8 enrichment on the CEBPE promoter in H929 and ARP‐1 cells after ZMYND8 knockdown. E) Relative mRNA expression of NSD2, ZMYND8, and CEBPE after NSD2 knockdown in H929 cells. Data in (E) are presented as mean ± SD, ** *p* < 0.01 by one‐way ANOVA test. F) Western blot analysis of NSD2, ZMYND8, CEBPE, and H3K36me2 expression following NSD2 knockdown in H929 cells. G) ChIP–qPCR analysis of ZMYND8 and H3K36me2 enrichment in the CEBPE promoter P3 region in H929 (left) and ARP‐1 (right) cells after NSD2 knockdown. Data in (B–D) and (G) are presented as mean ± SD, ** *p* < 0.01, *** *p* < 0.001 by two‐way ANOVA test; ns, nonsignificant. H) Schematic representation of the construction of ZMYND8‐PBP domain deletion mutants. I) Relative CEBPE/ZMYND8 mRNA levels in H929 cells transfected with ZMYND8 overexpression plasmids (ZMYND8‐WT, ΔPHD, ΔBRD, ΔPWWP, and F307A). Data in (I) are presented as mean ± SD, *** *p* < 0.001 by one‐way ANOVA test; ns, nonsignificant. J) Immunoblots showing ZMYND8 and CEBPE expression in H929 cells transfected with the EV, ZMYND8 wild‐type (WT), or ZMYND8 mutant plasmids. K) CCK‐8 assays in H929 cells transfected with ZMYND8 overexpression or vector plasmids. L) Coimmunoprecipitation assay with lysates extracted from ZMYND8‐KD H929 cells after transfection with the ZMYND8‐WT or ZMYND8‐F307A plasmid. An anti‐H3K36me2 antibody was applied for immunoprecipitation, and IgG served as a negative control. M) ChIP–reChIP analysis of relative ZMYND8 enrichment on the CEBPE promoter in ZMYND8‐KD H929 cells after transfection with the ZMYND8‐WT or ZMYND8‐F307A plasmid. An anti‐H3K36me2 antibody was used for the first immunoprecipitation. Data in (M) are presented as mean ± SD, *** *p* < 0.001 by two‐way ANOVA test. N) Western blot analysis of ZMYND8 and CEBPE expression in ZMYND8‐KD H929 cells transfected with the ZMYND8‐WT or ZMYND8‐F307A plasmid. O) CCK‐8 assays in ZMYND8‐KD H929 cells transfected with the ZMYND8‐WT or ZMYND8‐F307A plasmid. Data in (K) and (O) are presented as mean ± SD, ** *p* < 0.01 by two‐way ANOVA test. Fold enrichment was calculated relative to the IgG control in all ChIP–qPCR analysis.

Using the CUT&Tag data of MM cells, we observed that the ZMYND8 peaks were primarily enriched in the promoter region within 1 kb of the transcription start site (TSS) (70.88%) (Figure S7A,B, Supporting Information), whereas the H3K36me2 peaks were distributed primarily in the distal intergenic region (52.44%), followed by the promoter region within 1 kb of the TSS (26.83%) (Figure S7C,D, Supporting Information). These distribution characteristics indicate that both ZMYND8 and H3K36me2 have pivotal regulatory effects on the transcription of downstream target genes. CUT&Tag analyses also revealed that ZMYND8 overexpression did not lead to obvious changes in H3K36me2 enrichment at the CEBPE locus (Figure S7E, Supporting Information). Considering that H3K36 methylation is a dynamic process, we detected H3K36me1/2/3 levels in ZMYND8‐OE cells and found no significant alterations in H3K36me1/2/3 expression compared with that in vector controls (Figure S7F, Supporting Information). We designed six pairs of walking primers on the core promoter regions of CEBPE for ChIP–qPCR experiments to test the binding states of ZMYND8 and H3K38me2 to the CEBPE promoter (**Figure** **6**A). In H929 cells, ZMYND8 was substantially enriched in the P1–4 regions of the CEBPE promoter, and ZMYND8 overexpression significantly increased ZMYND8 enrichment in these regions, particularly in P3 (Figure 6B, left panel). Similar results were observed in ARP‐1 cells, although the level of ZMYND8 enrichment was lower than that observed in H929 cells (Figure 6B, right panel). H3K36me2 was enriched mainly in the P1–4 regions (particularly P3) in the CEBPE promoter in MM cells, which coincided exactly with the binding sites of ZMYND8, and no significant changes in the enrichment level of H3K36me2 were detected upon ZMYND8 overexpression (Figure 6C). ChIP–qPCR analysis further revealed that ZMYND8 enrichment was markedly decreased in the P3 region of the CEBPE promoter following ZMYND8 knockdown in H929 and ARP‐1 cells (Figure 6D). These findings imply that ZMYND8 binds directly to the CEBPE promoter and potentially interacts with H3K36me2.

To verify the dependence of ZMYND8 on H3K36me2 modification in the transcriptional regulation of CEBPE, we observed the corresponding changes by knocking down NSD2, a crucial H3K36me2 writer protein in MM cells. ZMYND8 mRNA expression did not change significantly after NSD2 knockdown, whereas CEBPE transcription was markedly downregulated in NSD2‐KD cells (Figure 6E and Figure S7G (Supporting Information)). Western blot analysis revealed that H3K36me2 levels were clearly reduced in MM cells following NSD2 knockdown. CEBPE protein expression was significantly decreased in H929 cells, but there was no obvious alteration in its expression in ARP‐1 cells (Figure 6F and Figure S7H (Supporting Information)). ChIP–qPCR analysis revealed that the enrichment of ZMYND8 and H3K36me2 decreased significantly in both the P1 and P3 regions of the CEBPE promoter after NSD2 knockdown in H929 cells, whereas the enrichment of ZMYND8 and H3K36me2 decreased only in the P3 region of ARP‐1 cells (Figure 6G and Figure S7I (Supporting Information)). Additionally, we found that the levels of histone H3K27me3 and H3K27ac were altered upon ZMYND8 overexpression, while there were no obvious changes in the levels of H3K9me3, H3K4me3, H3K4me1, H4K16ac, and H3K14ac between vector control and ZMYND8‐OE cells (Figure S7J, Supporting Information). We further performed ChIP experiments at the CEBPE promoter level and found that the enrichment levels of H3K27me3 and H3K27ac were low and did not change significantly after ZMYND8 overexpression (Figure S7K, Supporting Information). Together, these results suggest that ZMYND8 predominantly relies on its recognition of H3K36me2 to mediate the transcriptional activation of CEBPE, with a more pronounced effect observed in H929 cells harboring t(4;14).

To explore the structural details through which ZMYND8 recognizes H3K36me2 to regulate CEBPE expression, we constructed four deletion mutants on the basis of the ZMYND8 functional PBP domain, including PHD deletion (ΔPHD), BRD deletion (ΔBRD), PWWP deletion (ΔPWWP), and F307A point mutation (function loss of the PWWP domain) (Figure 6H). Compared with the wild‐type (WT), ZMYND8‐ΔPHD or ΔBRD did not obviously alter the relative CEBPE expression, whereas ZMYND8‐ΔPWWP or F307A led to a significant reduction in CEBPE transcription (Figure 6I and Figure S8A (Supporting Information)). Compared with those in ZMYND8‐WT, ΔPHD, and ΔBRD cells, the translational levels of CEBPE were evidently lower in ZMYND8‐ΔPWWP or F307A mutant cells (Figure 6J and Figure S8B (Supporting Information)). Compared with those of the ZMYND8‐ΔPWWP, F307A, and vector control groups, the proliferative capacities of MM cells were significantly lower in the ZMYND8‐WT, ΔPHD, and ΔBRD groups (Figure 6K and Figure S8C (Supporting Information)). Furthermore, coimmunoprecipitation experiments revealed that ZMYND8 could bind to H3K36me2 and that the ability of ZMYND8‐F307A to bind H3K36me2 was strikingly decreased compared with that of wild‐type ZMYND8 (Figure 6L and Figure S8D (Supporting Information)). ChIP–reChIP was performed via chromatin immunoprecipitation with an H3K36me2 antibody, followed by reimmunoprecipitation with a ZMYND8 antibody. The F307A mutation impaired the binding of the ZMYND8 protein to H3K36me2, leading to a decrease in the enrichment of ZMYND8 in the CEBPE promoter P3 region (Figure 6M and Figure S8E (Supporting Information)). The CUT&Tag data also revealed that the F307A mutation caused an obvious reduction in the enrichment level of ZMYND8 on the CEBPE promoter (Figure S8F, Supporting Information). Next, we stably overexpressed ZMYND8‐WT or ZMYND8‐F307A in ZMYND8‐KD cells. The decreased mRNA and protein levels of CEBPE caused by ZMYND8 knockdown were restored by ZMYND8‐WT but not by ZMYND8‐F307A in H929 and ARP‐1 cells (Figure 6N and Figure S8G,H (Supporting Information)). ZMYND8‐KD MM cells rescued with ZMYND8‐WT exhibited significantly weaker proliferation and cloning than those rescued with ZMYND8‐F307A (Figure 6O and Figure S8I,J (Supporting Information)). Furthermore, overexpression of ZMYND8‐WT, but not ZMYND8‐F307A, significantly increased the accumulation of unfolded or misfolded proteins through inhibiting the adaptive UPR (Figure S8K, Supporting Information). The immunofluorescence assay showed that overexpression of ZMYND8‐F307A failed to suppress the IRE1α expression in MM cells (Figure S8L, Supporting Information). The TEM results showed that under tunicamycin induction, the swelling degree of endoplasmic reticulum was milder in the ZMYND8‐F307A group than that in the ZMYND8‐WT group (Figure S8M, Supporting Information). Taken together, these results demonstrate that ZMYND8 interacts with H3K36me2 through its PWWP domain to regulate CEBPE expression, thereby affecting MM cell proliferation via regulating UPR.

### 2.7 ZMYND8 Overexpression Enhances the Sensitivity of MM Cells to PIs

**Figure 7:**
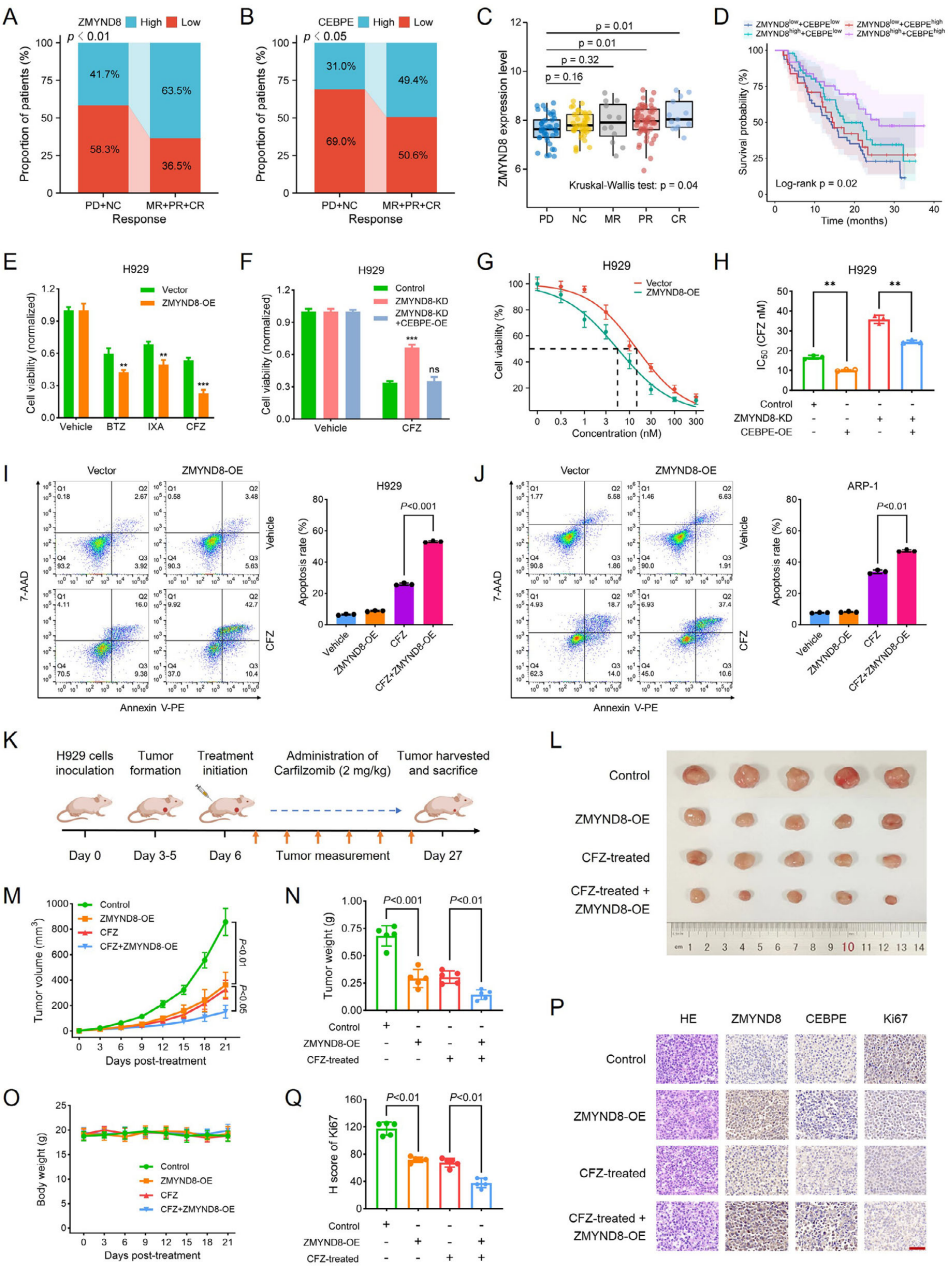
Upregulation of ZMYND8 expression increases the sensitivity of MM cells to CFZ treatment. A,B) Proportion of patients with high and low expression of ZMYND8 (A) and CEBPE (B) categorized by clinical response to PI therapy. Data in (A) and (B) were compared by Chi‐squared test. C) ZMYND8 expression levels in patients with different treatment responses in the GSE9782 dataset (*n* = 169). Data in (C) are presented as median ± IQR and analyzed by Kruskal–Wallis test with Dunn's posttest. D) Kaplan–Meier survival curve of OS for PI‐treated patients with MM based on both ZMYND8 and CEBPE expression. Patients were classified into four groups: ZMYND8^high^ and CEBPE^high^ (*n* = 37), ZMYND8^high^ and CEBPE^low^ (*n* = 52), ZMYND8^low^ and CEBPE^high^ (*n* = 31), and ZMYND8^low^ and CEBPE^low^ (*n* = 49). E) Cell viability was determined 36 h after treatment with DMSO (vehicle), 10 nm BTZ, 10 nm IXA, or 10 nm CFZ in H929 cells transfected with vector or ZMYND8‐OE plasmids. Data in (E) are presented as mean ± SD, ** *p* < 0.01, *** *p* < 0.001 by Student's *t*‐test. F) Cell viability was determined 48 h after treatment with DMSO or 10 nm CFZ in H929 cells treated with the negative control, ZMYND8‐KD, or ZMYND8‐KD + CEBPE‐OE. G) Comparison of the IC_50_ values of CFZ between the vector control and ZMYND8‐OE groups in H929 cells. H) IC_50_ values of CFZ in H929 cells treated with the negative control, ZMYND8‐KD, CEBPE‐OE, or ZMYND8‐KD + CEBPE‐OE. Data in (F) and (H) are presented as mean ± SD, ** *p* < 0.01, *** *p* < 0.001 by one‐way ANOVA test; ns, nonsignificant. I,J) Apoptosis analysis via flow cytometry (left panel) and quantification of the apoptotic rates (right panel) in normal or ZMYND8‐OE H929 (I) and ARP‐1 (J) cells treated with DMSO or 10 nm CFZ for 48 h. Data in (I) and (J) are presented as mean ± SD and analyzed by one‐way ANOVA test. K) Schematic diagram of in vivo drug administration. L–O) Images of tumor size (L), tumor volume (M), tumor weight (N), and mouse weight (O) in the experimental groups of mice. Data in (M) and (O) are presented as mean ± SD and analyzed by two‐way ANOVA test (*n* = 5). P) HE staining and IHC analysis of the indicated proteins in tumor xenografts excised from the mice in each group. The ruler length represents 50 µm. Q) *H*‐score analysis of the Ki67 protein in tumor xenografts excised from mice in the experimental groups. Data in (N) and (Q) are presented as mean ± SD and analyzed by one‐way ANOVA test (*n* = 5). Abbreviations: BTZ, bortezomib; CFZ, carfilzomib; CR, complete response; IC_50_, half‐maximal inhibitory concentration; IXA, ixazomib; MR, minimal response; NC, no change; OS, overall survival; PD, progressive disease; PI, proteasome inhibitor; PR, partial response.

Given the imperative role of ZMYND8 in MM progression, we investigated whether ZMYND8 has therapeutic significance in mainstream therapies for MM, such as PIs. We analyzed the therapeutic responses of 169 patients receiving bortezomib (BTZ) treatment via the GSE9782 dataset. There was a greater proportion of patients with low ZMYND8 or CEBPE expression in the nonresponsive group than in the responsive group (ZMYND8: 58.3% vs 36.5%, **Figure** **7**A; CEBPE: 69.0% vs 50.6%, Figure 7B). The ZMYND8 expression level tended to increase concomitantly with a gradual improvement in treatment response (Figure 7C). Patients with low ZMYND8 expression were more likely to have no response or progressive disease than were those with high ZMYND8 expression (61.3% vs 39.3%), and low CEBPE expression predicted a greater likelihood of no response or disease progression (57.4% vs 38.2%) (Figure S9A, Supporting Information). Following BTZ treatment, patients with both low ZMYND8 and CEBPE expression had the worst survival outcomes, whereas those with both high ZMYND8 and CEBPE expression had the best prognoses (Figure 7D).

We subsequently evaluated the effect of ZMYND8 expression on the sensitivity of MM cells to three clinically approved PIs: BTZ, CFZ, and ixazomib (IXA). The sensitivity of H929 and ARP‐1 cells to all three PIs significantly increased after ZMYND8 overexpression (Figure 7E and Figure S9B (Supporting Information)), whereas ZMYND8 knockdown conferred resistance to PIs in MM cells (Figure S9C, Supporting Information). Among these PIs, the therapeutic effect of CFZ was the most sensitive to changes in ZMYND8 expression. Therefore, CFZ was selected for subsequent experiments. The decreased sensitivity of ZMYND8‐KD MM cells to CFZ was reversed by simultaneous overexpression of CEBPE (Figure 7F and Figure S9D (Supporting Information)). Compared with those in the vector control, the half‐maximal inhibitory concentration (IC_50_) values of CFZ in ZMYND8‐OE cells were significantly lower (H929: 5.43 vs 14.49 nm; ARP‐1: 3.66 vs 10.74 nm), indicating improved sensitivity of these cells to CFZ treatment (Figure 7G and Figure S9E (Supporting Information)). CEBPE overexpression increased CFZ sensitivity in MM cells and effectively reversed the resistance to CFZ induced by ZMYND8 knockdown, as indicated by the corresponding IC_50_ values (Figure 7H and Figure S9F (Supporting Information)). Moreover, a significant increase in the rate of cellular apoptosis was observed in both H929 and ARP‐1 cells when ZMYND8 was overexpressed in combination with CFZ compared with that in cells treated with CFZ alone (Figure 7I,J). We confirmed that ZMYND8 still significantly upregulated CEBPE in MM cells when treated with CFZ (Figure S9G, Supporting Information). We also treated ZMYND8‐overexpressed MM cells with concentration gradients of CFZ and found that overexpression of ZMYND8 helped to reduce the administered amount of CFZ (Figure S9H, Supporting Information), which implies that upregulating ZMYND8 may favor the dose reduction of CFZ in patients with MM. In addition, our results showed that ZMYND8‐ΔPWWP or F307A mutations did not effectively enhance the sensitivity of MM cells to CFZ (Figure S9I,J, Supporting Information). Together, these findings suggest that ZMYND8 augments the sensitivity of MM cells to CFZ by regulating CEBPE expression.

To evaluate the in vivo therapeutic efficacy of CFZ upon ZMYND8 overexpression, we established xenograft tumor models derived from H929 cells. Tumor‐bearing mice were treated with intraperitoneal injections of CFZ (2 mg kg^−1^) for three weeks (Figure 7K). CFZ monotherapy exhibited moderate therapeutic efficacy against MM, and ZMYND8 overexpression significantly suppressed tumor proliferation and augmented the efficacy of CFZ in MM (Figure 7L–N). There were no significant changes in body weight among any of the experimental groups during the treatment period (Figure 7O). Similarly, we confirmed the elevated protein levels of both ZMYND8 and CEBPE in the ZMYND8‐OE and CFZ plus ZMYND8‐OE groups via IHC analysis (Figure 7P and Figure S9K (Supporting Information)). Additionally, Ki67 staining indicated that the combination of CFZ and ZMYND8‐OE significantly attenuated the proliferative capacity of MM tumor cells (Figure 7P,Q). Taken together, these findings indicate that ZMYND8 overexpression enhances the efficacy of CFZ in suppressing MM tumor growth.

## 3 Discussion

Altered expression of ZMYND8 is strongly associated with oncogenesis and tumor development.^[^ ^14^ ^]^ Elevated ZMYND8 expression has protumorigenic effects on colorectal cancer, hepatocellular carcinoma, and bladder cancer.^[^ ^21^, ^22^, ^24^, ^26^ ^]^ Conversely, low ZMYND8 expression promotes tumor malignancy in prostate cancer and nasopharyngeal carcinoma.^[^ ^17^, ^19^ ^]^ Thus, the function of ZMYND8 in different tumors remains controversial. In the present study, we reported that ZMYND8 was downregulated in MM tissues compared with normal or MGUS tissues and that low ZMYND8 expression was correlated with aggressive clinicopathological features. Patients with low ZMYND8 expression had worse OS and PFS than did those with high ZMYND8 expression. These clinical observations were further supported by in vitro and in vivo experiments. ZMYND8 overexpression significantly inhibited the proliferation and invasion of MM cells and effectively suppressed tumor growth in mice, whereas ZMYND8 knockdown had the opposite effects. These results illustrate the tumor‐suppressive role of ZMYND8 in MM progression.

We showed that the CEBPE‐mediated inhibition of ER stress is the primary pathway through which ZMYND8 affects MM cells. CEBPE is a member of the C/EBP family of transcription factors and is an indispensable regulator of granulocyte differentiation and myeloid cell development.^[^ ^27^, ^28^, ^29^ ^]^ CEBPE knockout mice exhibit impaired neutrophil production, which ultimately leads to myelodysplasia and premature mortality.^[^ ^30^ ^]^ Patients with CEBPE mutations are prone to neutrophil‐specific granule deficiency, profound neutropenia, and severe infections.^[^ ^31^, ^32^ ^]^ In another hematological malignancy, AML, low CEBPE expression is strongly correlated with poor clinical prognosis and a high relapse rate,^[^ ^33^ ^]^ and the repression of CEBPE expression can potentiate leukemogenesis.^[^ ^34^ ^]^ Consistent with its role as a tumor suppressor in AML, downregulation of CEBPE predicts unfavorable survival outcomes in patients with MM, and overexpression of CEBPE effectively inhibits MM cell proliferation. During granulopoiesis, CEBPE interacts with the retinoblastoma tumor suppressor protein to strengthen the CEBPE‐mediated transcription of myeloid‐specific genes, whereas CEBPE binds to E2F and represses E2F‐mediated transcriptional activity.^[^ ^35^, ^36^ ^]^ Moreover, CEBPE localizes to the promoters of a series of well‐established prognostic genes associated with AML survival.^[^ ^33^ ^]^ Here, our results suggest that CEBPE binds to specific sites on the promoters of crucial ER‐stress‐related genes, such as ERN1, ATF6, and XBP1, in MM cells and has negative regulatory relationships with these target genes, serving as a transcriptional repressor.

ER stress is important in the pathogenesis and treatment of myeloma. The survival of myeloma cells is heavily dependent on the UPR pathway because of the continuous overproduction of secretory proteins that induce ER stress.^[^ ^37^ ^]^ The IRE1α/XBP1s pathway is pivotal in regulating the adaptive UPR. IRE1α has been shown to promote the malignant growth of MM cells by facilitating adaptation to chronic ER stress through enhanced ER‐associated degradation capabilities.^[^ ^38^ ^]^ The strong dependency of MM cells on IRE1α/XBP1s signaling underscores this pathway as a vulnerability for the clinical treatment of MM. The development of safe and effective inhibitors of IRE1α for MM therapy is urgently needed.^[^ ^39^ ^]^ We found that ZMYND8 or CEBPE overexpression significantly inhibited IRE1α and XBP1s, which blocked the adaptive stress responses required for MM cell survival. Our findings support the notion that targeting prosurvival ER stress pathways through the ZMYND8/CEBPE/IRE1α axis has the potential to be a novel therapeutic direction in MM.

H3K36me2 is a crucial driver of myeloma progression; however, the epigenetic mechanisms underlying its regulation are poorly understood. Wang et al. proposed that the H3K36me2 reader protein HRP2 functions as a suppressor of BTZ resistance and inhibits the survival of MM cells by demethylating H3K27me3 in the context of t(4;14) abnormalities.^[^ ^40^ ^]^ Here, we identified and confirmed a new H3K36me2 reader, ZMYND8, that activates CEBPE expression at the promoter level by recognizing H3K36me2 in MM cells. H3K36me2 is considered a hallmark of the active transcriptional state^[^ ^41^ ^]^ and provides a structural framework for reader modules that recognize methylated sites through their dimethyl lysine‐binding motifs.^[^ ^10^, ^42^ ^]^ In t(4;14) MM cells such as H929 cells, high NSD2 expression leads to genome‐wide amplification of H3K36me2, providing a favorable scenario for binding interactions and transcriptional regulation of reader proteins.^[^ ^43^ ^]^ Intriguingly, in ARP‐1 cells lacking t(4;14), we also observed that ZMYND8 regulated CEBPE through its interaction with H3K36me2, although this effect was slightly attenuated compared with that in H929 cells. This finding implied that the recognition of H3K36me2 by ZMYND8 was not strongly affected by the presence of t(4;14) in MM cells.

Structurally, the PBP domain of ZMYND8 can simultaneously bind to histones and DNA to recruit itself to chromatin transcription sites, and mutations in this reader ensemble impair interactions between ZMYND8 and histones.^[^ ^44^ ^]^ In solid tumor cell lines, ZMYND8 selectively interacts with H3K36me2 via its PBP domain to regulate ATRA response genes, maintain epithelial phenotypes, and induce cell differentiation, resulting in tumor suppression.^[^ ^15^, ^18^, ^20^ ^]^ Our findings demonstrated that the PWWP domain of ZMYND8 is indispensable for recognizing H3K36me2 to regulate CEBPE transcription and that mutations within this domain not only impede the activation of CEBPE expression but also abolish the suppression of malignant phenotypes in MM cells. Further studies are needed to determine the binding constant between ZMYND8 and H3K36me2 through isothermal titration calorimetry, surface plasmon resonance, and microscale thermophoresis experiments. Importantly, we showed that ZMYND8 acts as a direct inhibitor of NSD2‐induced H3K36me2 signaling in MM, providing a unique molecular perspective for the inhibition of oncogenic H3K36me2 pathways.

In the era of novel drugs, PIs are used as fundamental chemotherapeutics for patients with MM. However, resistance to chemotherapy remains a major challenge in the clinical management of patients.^[^ ^45^, ^46^ ^]^ We found that the ZMYND8 expression level was positively correlated with the sensitivity of MM cells to treatment with PIs, particularly CFZ. Mechanistically, BTZ‐ and CFZ‐resistant MM cells exhibit increased activation of the IRE1α/XBP1s arm of the UPR pathways,^[^ ^47^ ^]^ which can be suppressed by ZMYND8. The ZMYND8/CEBPE axis leads to a substantial reduction in IRE1α levels, and IRE1α inhibition has been demonstrated to significantly increase the therapeutic efficacy of PIs.^[^ ^38^ ^]^ These findings provide a biological rationale for combining CFZ with the upregulation of ZMYND8 expression for the treatment of MM. Our future studies will focus on identifying and developing specific ZMYND8 agonists to investigate their potential effects on myeloma.

**Figure 8:**
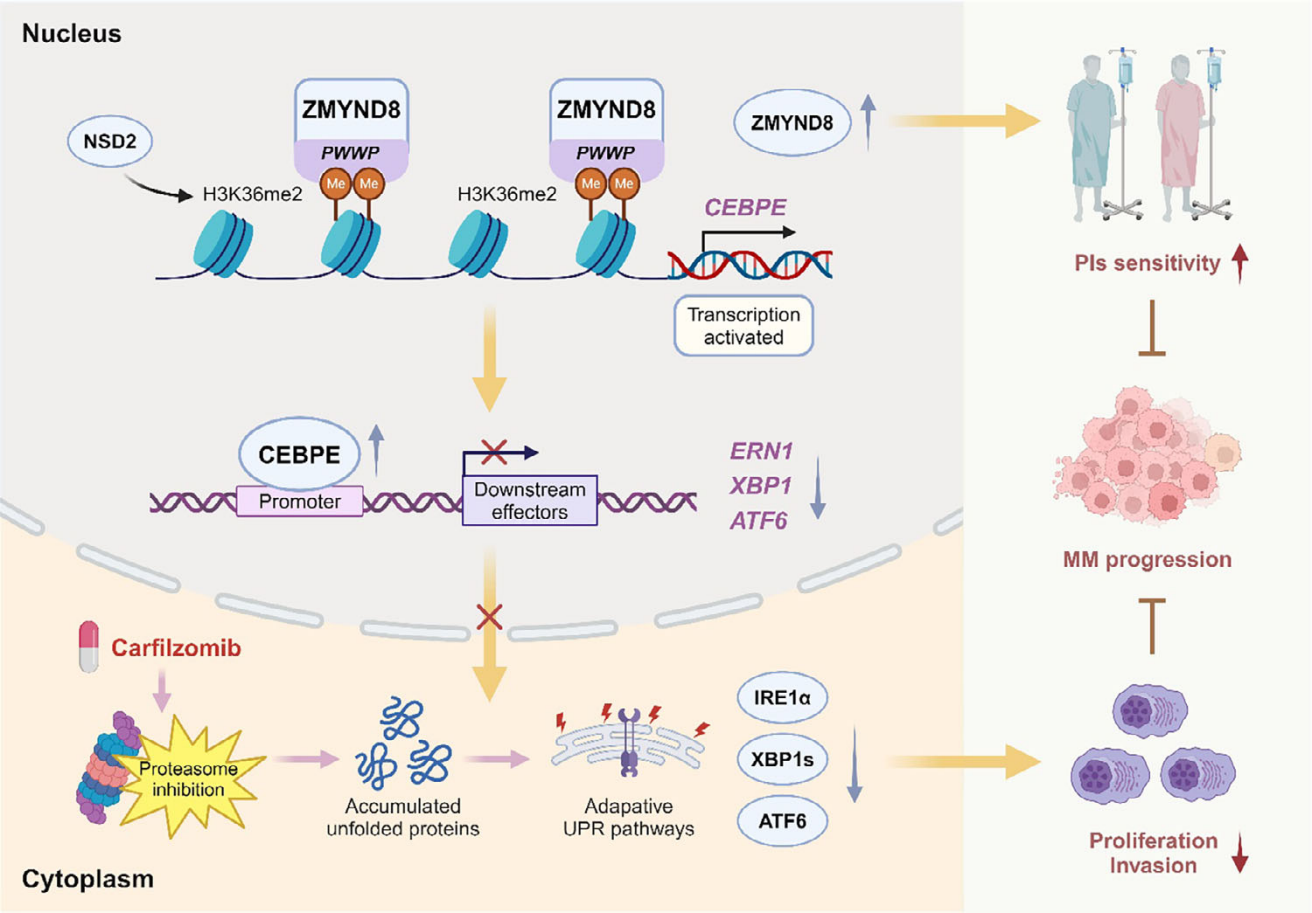
Illustration of the molecular mechanism by which ZMYND8 exerts tumor suppressor effects on MM cells. ZMYND8 recognizes H3K36me2 in the CEBPE promoter through its PWWP domain, leading to transcriptional activation of CEBPE, which represses the IRE1α pathway to inhibit cell proliferation and invasion, thereby attenuating disease progression and enhancing CFZ efficacy in patients with MM. The illustration was created with BioRender.com (accessed on 21 December 2024).

In conclusion, our study elucidated the precise mechanism by which ZMYND8 exerts its tumor‐suppressive effects in MM (**Figure** **8**). ZMYND8 activates the transcription of the tumor suppressor gene CEBPE by recognizing H3K36me2, thereby regulating ER stress and inhibiting the proliferation and invasion of MM cells. Clinically, ZMYND8 is a robust predictor of survival outcomes and PI sensitivity in patients with MM. Upregulating the expression of ZMYND8 under CFZ treatment effectively mitigated MM tumor progression. This study provides novel mechanistic insights into the role of ZMYND8 in MM biology and offers a promising therapeutic strategy for patients with MM.

## 4 Experimental Section

### Cell Lines and Cell Culture

Human MM cell lines (NCI‐H929, ARP‐1, RPMI‐8226, U266, and LP‐1) were maintained in RPMI‐1640 medium (Gibco, USA). Human embryonic kidney 293T (HEK293T) cells were maintained in Dulbecco's modified Eagle medium (Gibco, USA). Both culture media were supplemented with 10% fetal bovine serum (Biological Industries, Israel) and 1% penicillin–streptomycin (Beyotime Biotechnology, China). All the cells were cultured in a constant‐temperature incubator set at 37 °C with a humidified 5% CO_2_ atmosphere. The cell lines were routinely tested to exclude Mycoplasma infection and characterized by Genetic Testing Biotechnology (Suzhou, China) via short tandem repeat profiling.

### Lentiviral Transduction, siRNA Transfection, and Mutant Construction

To establish stable overexpression cells, the coding sequences of the target genes were cloned and inserted into the pCDH‐3×Flag‐GFP‐PuroR vector. To obtain stable ZMYND8‐knockdown cells, ZMYND8‐targeting shRNA and scrambled sequences were inserted into the pLKO.1‐PuroR vector. The lentiviruses of interest were obtained by the cotransfection of lentiviral expression constructs with packaging plasmids (psPAX2 and pMD2.G) into HEK293T cells via the use of Lipofectamine 3000 (Invitrogen, Carlsbad, CA, USA). After 48 h of transfection, the viral supernatants were collected for infection. Positive, stably transfected cells were screened with continuous 1 µg mL^−1^ puromycin treatment. siRNA targeting ZMYND8 was synthesized by KeyGen Biotech (Nanjing, China) and transiently transfected into cells via Lipofectamine 3000 (7.5 µL per 2 mL) with the 70% cell confluence. Site‐directed mutagenesis was performed via the Mut Express II Fast Mutagenesis Kit V2 (Vazyme Biotech, China). The point mutant F307A and the deletion mutant ZMYND8‐Δ were constructed following the manufacturer's instructions. The corresponding primer sequences are listed in Table S4 (Supporting Information).

### RNA Extraction and Quantitative Real‐Time PCR (qRT‐PCR) Analysis

Total cellular RNA was isolated using TRIzol reagent (Invitrogen, USA) and quantified via a NanoDrop ONE spectrophotometer (Thermo Scientific, USA). The extracted total RNA was reverse transcribed to cDNA via the HiScript II Q RT SuperMix for qPCR (+gDNA wiper) kit (Vazyme Biotech, China). Subsequently, qRT‐PCR was performed via AceQ qPCR SYBR Green Master Mix (Vazyme Biotech, China) on a StepOnePlus Real‐Time PCR System (Applied Biosystems, USA). The 2^−ΔΔCT^ method was used to calculate the relative mRNA expression values. The fold change in target gene expression was determined by normalization to that of GAPDH, and each independent sample was analyzed in triplicate. The sequences of the qRT‐PCR primers used are listed in Table S5 (Supporting Information).

### ChIP Assay

The cells were collected and cross‐linked with 1% formaldehyde for 15 min at room temperature. The cross‐linking reaction was terminated by adding 0.125 m glycine for 10 min. Chromatin extracts were subsequently sonicated to generate 200–500 bp DNA fragments. The chromatin mixture was incubated overnight with gentle rotation at 4 °C with 2 µg of specific primary antibodies or the negative control, IgG (Beyotime, China), followed by incubation with protein A‐Sepharose beads (GeneScript, China) at 4 °C for 2 h. After washing and elution of the immunoprecipitated complexes, the samples were digested with proteinase‐K (Invitrogen, USA), and the cross‐links were removed by heating at 65 °C overnight. DNA fragments were isolated and purified via phenol–chloroform extraction and ethanol precipitation. The precipitated DNA was dissolved in water and used for subsequent real‐time PCR. The sequences of primers used for ChIP–qPCR are listed in Table S6 (Supporting Information).

### Western Blot and Co‐IP

Total cellular proteins were extracted via cell lysis buffer for western blotting and IP (Beyotime, China). Core histone proteins were extracted from MM cells via acid extraction, as previously described.^[^ ^48^ ^]^ The protein concentration was quantitatively estimated via the bicinchoninic acid method. Proteins were separated via sodium dodecyl sulfate–polyacrylamide gel electrophoresis and transferred onto polyvinylidene difluoride membranes (Roche, Switzerland). The membranes were blocked with 5% nonfat milk in phosphate‐buffered saline (PBS)/0.1% Tween 20 for 1 h at room temperature and then incubated with primary antibodies at 4 °C overnight. On the second day after incubation with the appropriate secondary antibody, protein bands were detected via enhanced chemiluminescence reagents and visualized via a Tanon 5200 Imaging System (Tanon, Shanghai, China). For the coimmunoprecipitation experiments, 10% of the total protein lysate was used as the input. The remaining supernatant was aliquoted and mixed with negative control, IgG, or anti‐H3K36me2 antibody. The mixture was incubated on a rotator at 4 °C overnight and then immunoprecipitated with Protein A resin (GeneScript, China) at 4 °C for 3 h. Agarose beads were washed 3 times with NETN buffer, and pulled‐down proteins were detected via western blotting with the corresponding antibodies. Information on all the antibodies used is shown in Table  S7 (Supporting Information).

### CCK‐8 Assay

A CCK‐8 test was performed to assess cell viability and drug toxicity. Equal numbers of MM cells were inoculated into 96‐well plates at a volume of 100 µL. Every 24 h, 10 µL of CCK‐8 solution (Vazyme Biotech, China) was added to each well, and the plates were incubated at 37 °C for 2 h in the dark. The absorbance was measured at 450 nm via a Safire microplate reader (Tecan, Switzerland). For in vitro drug studies, PIs (bortezomib, carfilzomib, and ixazomib) were purchased from TargetMol (USA), and cell viability was measured after drug treatment.

### Methylcellulose Colony Formation Assay

Cell proliferation was tested via a colony‐forming unit assay using MethoCult H4100 Optimum (Stem Cell Tech, Vancouver, Canada). The cells were mixed with complete RPMI‐1640 medium in six‐well plates supplemented with methylcellulose. After 12 days of culture in 5% CO_2_ at 37 °C in a humidified incubator, the colony numbers were scored under an inverted microscope. All the wells were independently replicated 3 times.

### Apoptosis Assay by Flow Cytometry

Cell apoptosis was determined via an Annexin V‐PE/7‐AAD Apoptosis Detection Kit (Vazyme Biotech, China), and apoptotic rates were analyzed via flow cytometry. Briefly, cells (2 × 10^5^) were collected and washed twice with 1× cold PBS. Each sample was resuspended in 500 µL of binding buffer and stained with 5 µL of Annexin V‐PE and 5 µL of 7‐AAD. The cell suspension was then protected from light and incubated at room temperature for 10 min. All stained samples were tested via an Attune NxT flow cytometer (Thermo Fisher Scientific) within 1 h. The Annexin V‐PE(+)/7‐AAD(−) and Annexin V‐PE(+)/7‐AAD(+) subsets were considered apoptotic. Flow cytometry data analysis was performed via FlowJo software (version 10).

### Transwell Invasion Assay

Cell invasiveness was assessed via 8 µm pore size Transwell chambers (Corning Incorporated, NY, USA). The Matrigel matrix (Corning) was diluted in serum‐free medium and added to the upper chamber prior to plating the cells. After the cells were cultured in serum‐free RPMI‐1640 medium overnight, 2 × 10^4^ cells were added to the upper compartment of the Transwell chamber, whereas the lower compartment was filled with serum‐containing medium as a chemoattractant. After 24 h of incubation, the invasive cells were harvested from the lower chamber and counted via flow cytometry.

### Immunofluorescence

MM cells were cultured on glass slides and fixed with 4% paraformaldehyde for 15 min at room temperature. The cells were blocked with PBS containing 2% bovine serum albumin for 60 min after fixing on the slides. Blocking solution was then removed, and the cells were incubated with specific primary and secondary antibodies for 60 min each in a dark and humidified environment. After washing with PBS thrice, nuclei were stained with 4′,6‐diamidino‐2‐phenylindole for 10 min. Immunofluorescence images were captured using a fluorescence microscope (Olympus, USA).

### TEM

Cells were collected and fixed with 2.5% glutaraldehyde for 20 min at room temperature. After rinsing 3 times, the samples were postfixed with 1% osmium acid fixative solution for 2 h. Samples were then dehydrated through a gradient of ethanol concentrations and embedded in EPON812. After solidification, ultrathin sections of the samples were double‐stained with 2% uranyl acetate and lead citrate. The prepared samples were observed under a Hitachi HT7800 transmission electron microscope operating at 80 kV.

### Clinical Specimens and IHC Staining

Human MM samples were obtained from Nanjing Drum Tower Hospital, China. The collection of clinical samples was approved by the Medical Ethics Committee of the Affiliated Drum Tower Hospital of Nanjing University Medical School, China (approval number: 2023‐043‐02). All patients signed an informed consent agreement prior to sample collection. The paraffin‐embedded bone marrow sections were dewaxed, subjected to antigen retrieval, and blocked. The tissue slides were incubated with specific primary antibodies overnight at 4 °C, followed by the addition of horseradish‐peroxidase‐conjugated secondary antibodies the following day. The sections were visualized via diaminobenzidine and counterstained with hematoxylin and eosin. Each IHC staining result was independently evaluated by two experienced pathologists blinded to sample information. The staining intensity was scored on the following scale: negative (0), weak (1), moderate (2), and strong (3). An *H*‐score of 0–300 was obtained by multiplying the intensity score and percentage of cells with the corresponding score.

### Xenograft Models and In Vivo Drug Treatment

To establish xenograft MM tumor models, four‐week‐old female BALB/c nude mice were obtained from GemPharmatech Co., Ltd. (Nanjing, China). All experimental procedures involving animals were reviewed and approved by the Animal Ethics and Welfare Committee of Nanjing University, China (approval number: IACUC‐2302005). H929 cells were transduced with the corresponding lentiviruses to establish stable cell lines for in vivo functional validation. Next, 1 × 10^7^ transfected H929 cells were injected subcutaneously into the right side of each mouse (five mice per group). For in vivo drug studies, tumor‐bearing mice were randomly assigned to different treatment groups when subcutaneous xenografts were palpable. CFZ (2 mg kg^−1^) was administered intraperitoneally every two days. The body weights and tumor volumes were recorded every three days. The tumor volume was measured using a Vernier caliper and calculated via the following formula: tumor volume (mm^3^) = 0.5 × length × width^2^. The mice were euthanized via cervical dislocation, and the tumor tissues were harvested for analysis.

### RNA‐seq and CUT&Tag Analysis

To determine the potential target genes regulated by ZMYND8, H929 cells stably overexpressing the control vector or ZMYND8 were subjected to transcriptome sequencing. Quality control was performed via the Agilent 2100 Bioanalyzer after the cDNA libraries were constructed. Sequencing was performed on a NovaSeq 6000 platform (Illumina) in paired‐end, 150 bp mode. Differential expression analysis was performed via the DESeq package in R. The screening criteria for DEGs were a fold change >2 and a *p* value <0.05. A CUT&T assay was performed to determine the binding profiles of ZMYND8 and H3K36me2 in H929 cells. Library construction was performed via a CUT&Tag Assay Kit (pAG‐Tn5) for Illumina (ABclonal, Wuhan, China), following the recommended protocol. After quantification and quality control, the DNA libraries were sequenced on an Illumina NovaSeq 6000 platform. Downstream processing of sequencing data was conducted via bioinformatics analysis, and read distributions were visualized via Integrative Genomics Viewer software. The raw sequence data reported in this paper were deposited in the Genome Sequence Archive (Genomics, Proteomics & Bioinformatics, 2021) at the National Genomics Data Center (Nucleic Acids Res, 2022), China National Center for Bioinformation/Beijing Institute of Genomics, Chinese Academy of Sciences (GSA‐Human: HRA004092 and HRA006806), and were publicly accessible at https://ngdc.cncb.ac.cn/gsa‐human.^[^ ^49^, ^50^ ^]^

### Gene Expression Profiling

The MM‐related microarray and RNA‐seq data used in this study were obtained from the Gene Expression Omnibus database (https://www.ncbi.nlm.nih.gov/geo). Clinical, treatment, and follow‐up information was extracted from the corresponding MM datasets. The expression value of each gene was computed via a robust multiarray average algorithm. Relative mRNA expression levels were log‐transformed via the log_2_ function. For the survival analysis of ZMYND8 and CEBPE, the optimal cutoff expression values were determined via the Survminer package in R to divide patients into high‐ and low‐risk subgroups.

### Statistical Analysis

All statistical analyses were performed via GraphPad Prism (version 10.1.2) and R software (version 4.1.1). The normality of continuous variables was assessed via the Shapiro–Wilk test. The results were presented as the mean ± standard deviation (SD) unless otherwise stated. Comparisons of continuous data between the two groups were performed using Student's *t*‐test or Wilcoxon rank‐sum test. Multiple group comparisons were performed via one‐way analysis of variance (ANOVA) or Kruskal–Wallis test. The Chi‐squared test was used to compare categorical variables. The Kaplan–Meier method and log‐rank test were used for survival analysis. Correlation analysis was conducted via Spearman's rank correlation test. A two‐tailed *p* value < 0.05 was considered to indicate statistical significance.

## Conflict of Interest

The authors declare no conflict of interest.

## Author Contributions

J.X., X.D., and Y.P. contributed equally to this work. J.X. performed the research and wrote the paper. Y.P., J.Y., P.X., F.L., S.Z., L.C., X.M., K.W., M.X., W.L., and D.C.S.H. assisted with the data analysis and experiments. X.D., Q.Z., and B.C. supervised the project and revised the paper. All the authors have read and approved the final version of this paper.

## Supporting information



Supporting Information

## Data Availability

The datasets used and/or analyzed during the current study are available from the corresponding author upon reasonable request.

## References

[1] S. K. Kumar , V. Rajkumar , R. A. Kyle , M. van Duin , P. Sonneveld , M. V. Mateos , F. Gay , K. C. Anderson , Nat. Rev. Dis. Primers 2017, 3, 17046. 28726797 10.1038/nrdp.2017.46

[2] R. L. Siegel , A. N. Giaquinto , A. Jemal , CA‐Cancer J. Clin. 2024, 74, 12. 38230766 10.3322/caac.21820

[3] A. J. Cowan , D. J. Green , M. Kwok , S. Lee , D. G. Coffey , L. A. Holmberg , S. Tuazon , A. K. Gopal , E. N. Libby , JAMA, J. Am. Med. Assoc. 2022, 327, 464. 10.1001/jama.2022.0003 35103762

[4] R. A. Elnair , S. A. Holstein , Drugs 2021, 81, 825. 33871818 10.1007/s40265-021-01514-0

[5] A. Kalff , A. Spencer , Blood Cancer J. 2012, 2, 89. 10.1038/bcj.2012.37 PMC3461707 22961061

[6] S. Sato , W. Kamata , S. Okada , Y. Tamai , Int. J. Hematol. 2021, 113, 207. 32949373 10.1007/s12185-020-03005-6

[7] E. Martinez‐Garcia , R. Popovic , D. J. Min , S. M. Sweet , P. M. Thomas , L. Zamdborg , A. Heffner , C. Will , L. Lamy , L. M. Staudt , D. L. Levens , N. L. Kelleher , J. D. Licht , Blood 2011, 117, 211. 20974671 10.1182/blood-2010-07-298349 PMC3037745

[8] R. Popovic , E. Martinez‐Garcia , E. G. Giannopoulou , Q. Zhang , Q. Zhang , T. Ezponda , M. Y. Shah , Y. Zheng , C. M. Will , E. C. Small , Y. Hua , M. Bulic , Y. Jiang , M. Carrara , R. A. Calogero , W. L. Kath , N. L. Kelleher , J. P. Wang , O. Elemento , J. D. Licht , PLoS Genet. 2014, 10, 1004566. 10.1371/journal.pgen.1004566 PMC4154646 25188243

[9] A. J. Kuo , P. Cheung , K. Chen , B. M. Zee , M. Kioi , J. Lauring , Y. Xi , B. H. Park , X. Shi , B. A. Garcia , W. Li , O. Gozani , Mol. Cell 2011, 44, 609. 22099308 10.1016/j.molcel.2011.08.042 PMC3222870

[10] C. A. Musselman , M. E. Lalonde , J. Cote , T. G. Kutateladze , Nat. Struct. Mol. Biol. 2012, 19, 1218. 23211769 10.1038/nsmb.2436 PMC3645987

[11] H. Wen , X. Shi , Cancer Treat. Res. 2023, 190, 245. 38113004 10.1007/978-3-031-45654-1_8 PMC11395558

[12] I. O. Torres , D. G. Fujimori , Curr. Opin. Struct. Biol. 2015, 35, 68. 26496625 10.1016/j.sbi.2015.09.007 PMC4688207

[13] K. Hyun , J. Jeon , K. Park , J. Kim , Exp. Mol. Med. 2017, 49, 324. 10.1038/emm.2017.11 PMC6130214 28450737

[14] Y. Chen , Y. H. Tsai , S. H. Tseng , Molecules 2021, 26, 1083. 33670804 10.3390/molecules26041083 PMC7923094

[15] S. Adhikary , S. Sanyal , M. Basu , I. Sengupta , S. Sen , D. K. Srivastava , S. Roy , C. Das , J. Biol. Chem. 2016, 291, 2664. 26655721 10.1074/jbc.M115.679985 PMC4742736

[16] F. Gong , K. M. Miller , Cell Cycle 2018, 17, 414. 29393731 10.1080/15384101.2017.1376150 PMC5927707

[17] N. Li , Y. Li , J. Lv , X. Zheng , H. Wen , H. Shen , G. Zhu , T. Y. Chen , S. S. Dhar , P. Y. Kan , Z. Wang , R. Shiekhattar , X. Shi , F. Lan , K. Chen , W. Li , H. Li , M. G. Lee , Mol. Cell 2016, 63, 470. 27477906 10.1016/j.molcel.2016.06.035 PMC4975651

[18] M. Basu , I. Sengupta , M. W. Khan , D. K. Srivastava , P. Chakrabarti , S. Roy , C. Das , Biochem. J. 2017, 474, 1919. 28432260 10.1042/BCJ20170223

[19] J. Chen , J. Liu , X. Chen , Y. Li , Z. Li , C. Shen , K. Chen , X. Zhang , Cancer Manage. Res. 2019, 11, 7835. 10.2147/CMAR.S210305 PMC6713802 31692588

[20] S. Mukherjee , S. Sen , S. Adhikary , A. Sengupta , P. Mandal , D. Dasgupta , P. Chakrabarti , S. Roy , C. Das , J. Biosci. 2020, 45, 2. 31965980

[21] S. Choi , K. W. Lee , H. H. Koh , S. Park , S. Y. Yeo , J. W. Joh , M. S. Choi , S. H. Kim , C. K. Park , S. Y. Ha , J. Cancer Res. Clin. Oncol. 2021, 147, 3517. 34462784 10.1007/s00432-021-03768-3 PMC11802057

[22] C. Dou , H. Mo , T. Chen , J. Liu , Y. Zeng , S. Li , C. Guo , C. Zhang , Pathol., Res. Pract. 2021, 219, 153345. 33517164 10.1016/j.prp.2021.153345

[23] Q. Pan , S. Zhong , H. Wang , X. Wang , N. Li , Y. Li , G. Zhang , H. Yuan , Y. Lian , Q. Chen , Y. Han , J. Guo , Q. Liu , T. Qiu , J. Jiang , Q. Li , M. Tan , H. Yin , J. Peng , Y. Xiao , J. Qin , Mol. Cell 2021, 81, 2736. 33932349 10.1016/j.molcel.2021.04.009

[24] F. Qiu , Y. Jin , J. Pu , Y. Huang , J. Hou , X. Zhao , Y. Lu , Exp. Cell Res. 2021, 407, 112807. 34487730 10.1016/j.yexcr.2021.112807

[25] Z. Cao , K. A. Budinich , H. Huang , D. Ren , B. Lu , Z. Zhang , Q. Chen , Y. Zhou , Y. H. Huang , F. Alikarami , M. C. Kingsley , A. K. Lenard , A. Wakabayashi , E. Khandros , W. Bailis , J. Qi , M. P. Carroll , G. A. Blobel , R. B. Faryabi , K. M. Bernt , S. L. Berger , J. Shi , Mol. Cell 2021, 81, 3604. 34358447 10.1016/j.molcel.2021.07.018 PMC8932643

[26] J. Chen , Q. He , P. Wu , J. Fu , Y. Xiao , K. Chen , D. Xie , X. Zhang , Cancer Biomarkers 2020, 28, 201. 32224527 10.3233/CBM-191261 PMC12662352

[27] A. M. Chumakov , I. Grillier , E. Chumakova , D. Chih , J. Slater , H. P. Koeffler , Mol. Cell. Biol. 1997, 17, 1375. 9032264 10.1128/mcb.17.3.1375 PMC231862

[28] R. Morosetti , D. J. Park , A. M. Chumakov , I. Grillier , M. Shiohara , A. F. Gombart , T. Nakamaki , K. Weinberg , H. P. Koeffler , Blood 1997, 90, 2591. 9326225

[29] T. Akagi , N. H. Thoennissen , A. George , G. Crooks , J. H. Song , R. Okamoto , D. Nowak , A. F. Gombart , H. P. Koeffler , PLoS One 2010, 5, 15419. 10.1371/journal.pone.0015419 PMC2972224 21072215

[30] R. Yamanaka , C. Barlow , J. Lekstrom‐Himes , L. H. Castilla , P. P. Liu , M. Eckhaus , T. Decker , A. Wynshaw‐Boris , K. G. Xanthopoulos , Proc. Natl. Acad. Sci. USA 1997, 94, 13187. 9371821 10.1073/pnas.94.24.13187 PMC24284

[31] A. F. Gombart , M. Shiohara , S. H. Kwok , K. Agematsu , A. Komiyama , H. P. Koeffler , Blood 2001, 97, 2561. 11313242 10.1182/blood.v97.9.2561

[32] A. Z. Banday , A. Kaur , T. Akagi , D. Bhattarai , M. Muraoka , D. Dev , J. Das , M. U. S. Sachdeva , I. Karmakar , K. Arora , G. Kaur , V. Pandiarajan , A. K. Jindal , T. Wada , H. P. Koeffler , D. Suri , J. Ahluwalia , H. Kanegane , P. Bhatia , A. Rawat , S. Singh , J. Clin. Immunol. 2022, 42, 1434. 35726044 10.1007/s10875-022-01304-7

[33] K. Li , Y. Du , D. Q. Wei , F. Zhang , J. Transl. Med. 2019, 17, 188. 31164135 10.1186/s12967-019-1944-x PMC6549322

[34] S. M. Park , H. Cho , A. M. Thornton , T. S. Barlowe , T. Chou , S. Chhangawala , L. Fairchild , J. Taggart , A. Chow , A. Schurer , A. Gruet , M. D. Witkin , J. H. Kim , E. M. Shevach , A. Krivtsov , S. A. Armstrong , C. Leslie , M. G. Kharas , Cell Stem Cell 2019, 24, 153. 30472158 10.1016/j.stem.2018.10.016 PMC6602096

[35] S. Gery , A. F. Gombart , Y. K. Fung , H. P. Koeffler , Blood 2004, 103, 828. 12947005 10.1182/blood-2003-01-0159

[36] K. Theilgaard‐Monch , S. Pundhir , K. Reckzeh , J. Su , M. Tapia , B. Furtwangler , J. Jendholm , J. S. Jakobsen , M. S. Hasemann , K. J. Knudsen , J. B. Cowland , A. Fossum , E. Schoof , M. B. Schuster , B. T. Porse , Nat. Commun. 2022, 13, 3595. 35739121 10.1038/s41467-022-31332-1 PMC9225994

[37] S. White‐Gilbertson , Y. Hua , B. Liu , Front. Genet. 2013, 4, 109. 23781234 10.3389/fgene.2013.00109 PMC3678081

[38] J. M. Harnoss , A. Le Thomas , A. Shemorry , S. A. Marsters , D. A. Lawrence , M. Lu , Y. A. Chen , J. Qing , K. Totpal , D. Kan , E. Segal , M. Merchant , M. Reichelt , H. Ackerly Wallweber , W. Wang , K. Clark , S. Kaufman , M. H. Beresini , S. T. Laing , W. Sandoval , M. Lorenzo , J. Wu , J. Ly , T. De Bruyn , A. Heidersbach , B. Haley , A. Gogineni , R. M. Weimer , D. Lee , M. G. Braun , et al., Proc. Natl. Acad. Sci. USA 2019, 116, 16420. 31371506 10.1073/pnas.1906999116 PMC6697881

[39] N. Mimura , M. Fulciniti , G. Gorgun , Y. T. Tai , D. Cirstea , L. Santo , Y. Hu , C. Fabre , J. Minami , H. Ohguchi , T. Kiziltepe , H. Ikeda , Y. Kawano , M. French , M. Blumenthal , V. Tam , N. L. Kertesz , U. M. Malyankar , M. Hokenson , T. Pham , Q. Zeng , J. B. Patterson , P. G. Richardson , N. C. Munshi , K. C. Anderson , Blood 2012, 119, 5772. 22538852 10.1182/blood-2011-07-366633 PMC3382937

[40] J. Wang , X. Zhu , L. Dang , H. Jiang , Y. Xie , X. Li , J. Guo , Y. Wang , Z. Peng , M. Wang , J. Wang , S. Wang , Q. Li , Y. Wang , Q. Wang , L. Ye , L. Zhang , Z. Liu , J. Clin. Invest. 2022, 132, e149526. 35166240 10.1172/JCI149526 PMC8843744

[41] J. Li , J. H. Ahn , G. G. Wang , Cell. Mol. Life Sci. 2019, 76, 2899. 31147750 10.1007/s00018-019-03144-y PMC11105573

[42] D. J. Patel , Z. Wang , Annu. Rev. Biochem. 2013, 82, 81. 23642229 10.1146/annurev-biochem-072711-165700 PMC4696766

[43] G. LeRoy , O. Oksuz , N. Descostes , Y. Aoi , R. A. Ganai , H. O. Kara , J. R. Yu , C. H. Lee , J. Stafford , A. Shilatifard , D. Reinberg , Sci. Adv. 2019, 5, aay3068. 10.1126/sciadv.aay3068 PMC6774727 31616795

[44] P. Savitsky , T. Krojer , T. Fujisawa , J. P. Lambert , S. Picaud , C. Y. Wang , E. K. Shanle , K. Krajewski , H. Friedrichsen , A. Kanapin , C. Goding , M. Schapira , A. Samsonova , B. D. Strahl , A. C. Gingras , P. Filippakopoulos , Cell Rep. 2016, 17, 2724. 27926874 10.1016/j.celrep.2016.11.014 PMC5177622

[45] P. Robak , I. Drozdz , J. Szemraj , T. Robak , Cancer Treat. Rev. 2018, 70, 199. 30245231 10.1016/j.ctrv.2018.09.001

[46] C. T. Wallington‐Beddoe , M. Sobieraj‐Teague , B. J. Kuss , S. M. Pitson , Br. J. Haematol. 2018, 182, 11. 29676460 10.1111/bjh.15210

[47] T. Kubicki , K. Bednarek , M. Kostrzewska‐Poczekaj , M. Luczak , K. Lewandowski , L. Gil , M. Jarmuz‐Szymczak , D. Dytfeld , Am. J. Cancer Res. 2022, 12, 3280. 35968359 PMC9360248

[48] D. Shechter , H. L. Dormann , C. D. Allis , S. B. Hake , Nat. Protoc. 2007, 2, 1445. 17545981 10.1038/nprot.2007.202

[49] T. Chen , X. Chen , S. Zhang , J. Zhu , B. Tang , A. Wang , L. Dong , Z. Zhang , C. Yu , Y. Sun , L. Chi , H. Chen , S. Zhai , Y. Sun , L. Lan , X. Zhang , J. Xiao , Y. Bao , Y. Wang , Z. Zhang , W. Zhao , Genomics, Proteomics Bioinf. 2021, 19, 578. 10.1016/j.gpb.2021.08.001 PMC9039563 34400360

[50] C.‐N. Members , Nucleic Acids Res. 2022, 50, D27. 34718731 10.1093/nar/gkab951 PMC8728233

