# Supplementary material for: ZMYND8 Reads H3K36me2 to Activate CEBPE Transcription and Suppress Multiple Myeloma Progression through the Inhibition of Adaptive UPR Pathways
Source: Adv Sci (Weinh). 2025 May 10;12(20):2409219. doi: 10.1002/advs.202409219 (PMC12120720; doi:10.1002/advs.202409219)
Supplement: Supplementary file 1 — Supporting Information [file ADVS-12-2409219-s001.pdf]

ADVANCED SCIENCE
Open Access

Supporting Information

for *Adv. Sci.*, DOI 10.1002/advs.202409219

ZMYND8 Reads H3K36me2 to Activate CEBPE Transcription and Suppress Multiple Myeloma Progression through the Inhibition of Adaptive UPR Pathways

Jiaxuan Xu, Xiaoqing Dong, Yue Peng, Jie Yan, Peipei Xu, Feiyu Li, Suwen Zhang, Lanxin Chen, Xingjun Meng, Kangning Wang, Mengying Xing, Wenyang Li, David C. S. Huang, Quan Zhao* and Bing Chen**

Supporting information

ZMYND8 reads H3K36me2 to activate CEBPE transcription and suppress multiple myeloma progression through the inhibition of adaptive UPR pathways

Jiaxuan Xu, Xiaoqing Dong, Yue Peng, Jie Yan, Peipei Xu, Feiyu Li, Suwen Zhang, Lanxin Chen, Xingjun Meng, Kangning Wang, Mengying Xing, Wenyang Li, David C. S. Huang, Quan Zhao*, Bing Chen**

File list

1. Figure S1

2. Figure S2

3. Figure S3

4. Figure S4

5. Figure S5

6. Figure S6

7. Figure S7

8. Figure S8

9. Figure S9

10. Table S1

11. Table S2

12. Table S3

13. Table S4

14. Table S5

15. Table S6

16. Table S7

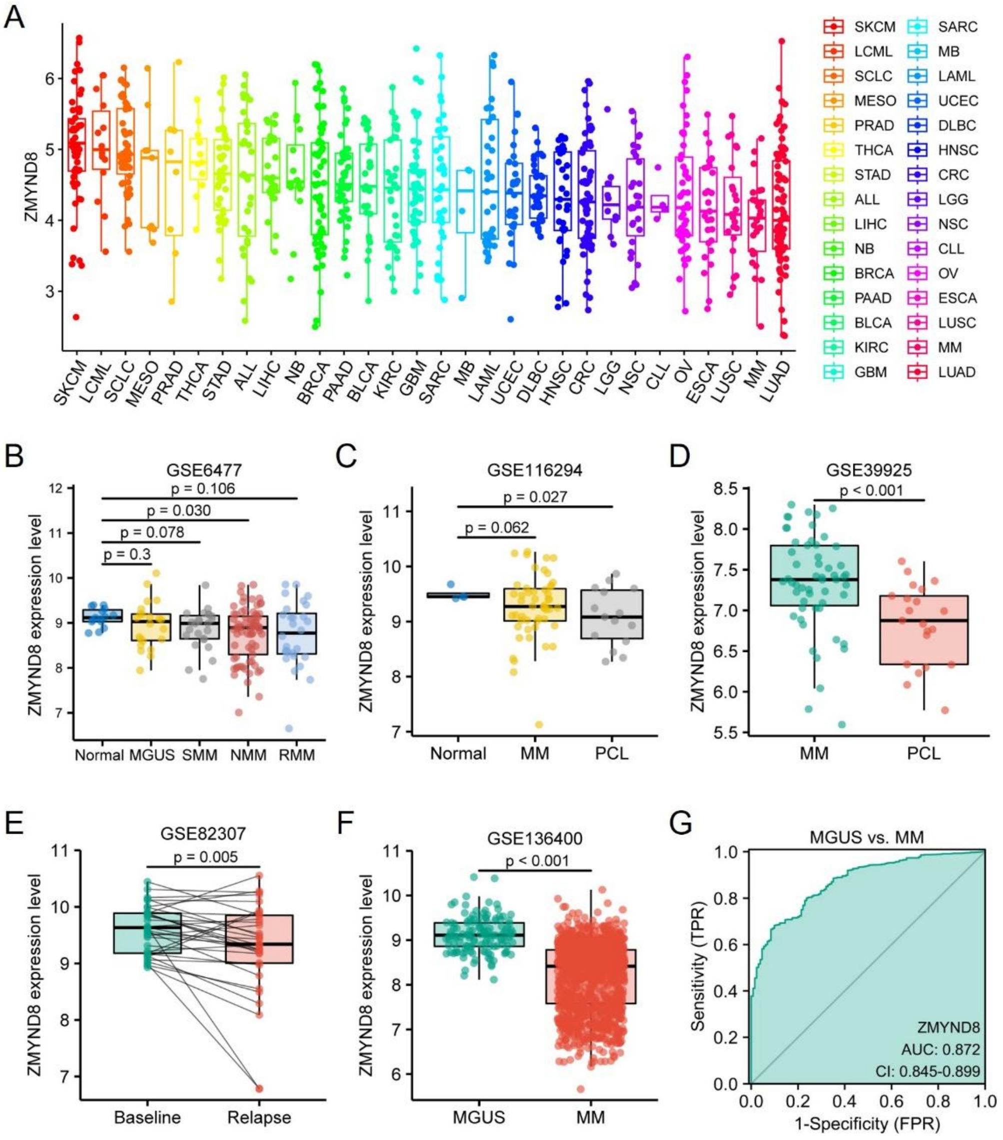

Figure S1. (A) Pan-cancer analysis of ZMYND8 mRNA expression among human tumor cell lines using the CCLE database (https://sites.broadinstitute.org/ccle). Cancer types were arranged in order from high to low ZMYND8 expression. (B) Expression levels of ZMYND8 during the disease progression of MM in the GSE6477 dataset. (C, D) Expression levels of ZMYND8 in MM and PCL patients from the GSE116294 (C) and GSE39925 (D) datasets. (E) Comparison of ZMYND8 expression in paired MM samples at baseline and relapse in the GSE82307 dataset. (F, G) Expression levels (F) and diagnostic ROC curve analysis (G) of ZMYND8 in MM (n = 1293) versus MGUS (n = 131) in the GSE136400 dataset. Data in B–F are visualized with boxplots and presented as median ± interquartile range (IQR). MGUS, monoclonal gammopathy of undetermined significance; SMM, smoldering multiple myeloma; NMM, newly diagnosed MM; RMM, relapsed MM; PCL, plasma cell leukemia; AUC, area under the curve.

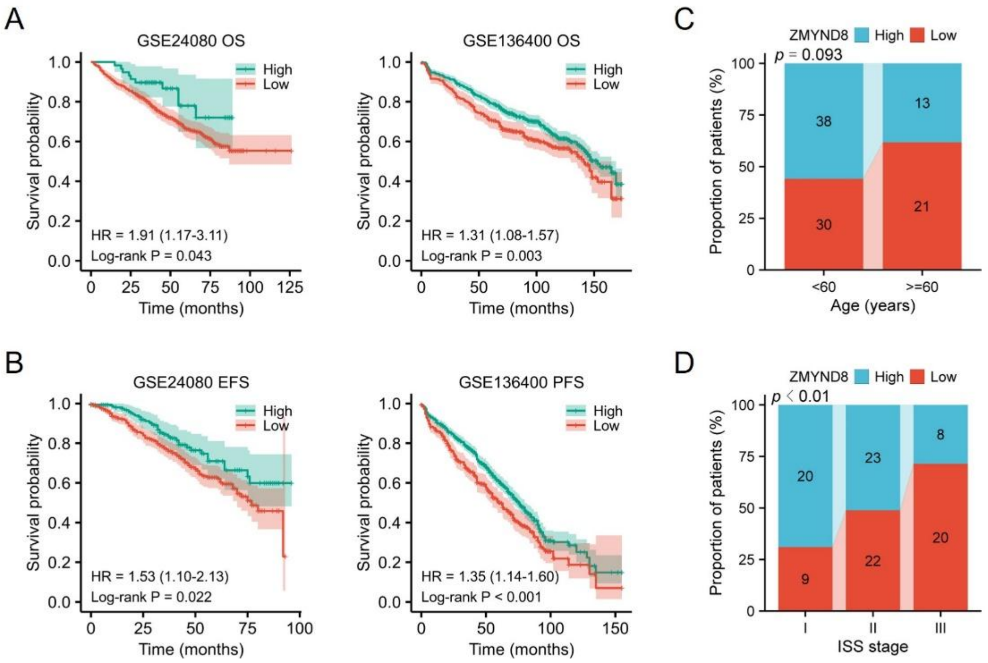

Figure S2. (A) Kaplan-Meier curves of OS for patients with high and low expression of ZMYND8 in the GSE24080 and GSE136400 datasets. (B) Kaplan-Meier curves of EFS (left panel) and PFS (right panel) for patients with high and low ZMYND8 expression in the GSE24080 and GSE136400 datasets. (C, D) The proportions of patients with high and low expression of ZMYND8 in different subgroups according to age (C) and ISS stage (D). Data in C and D were compared by Chi-squared test. OS, overall survival; EFS, event-free survival; PFS, progression-free survival; ISS, International Staging System.

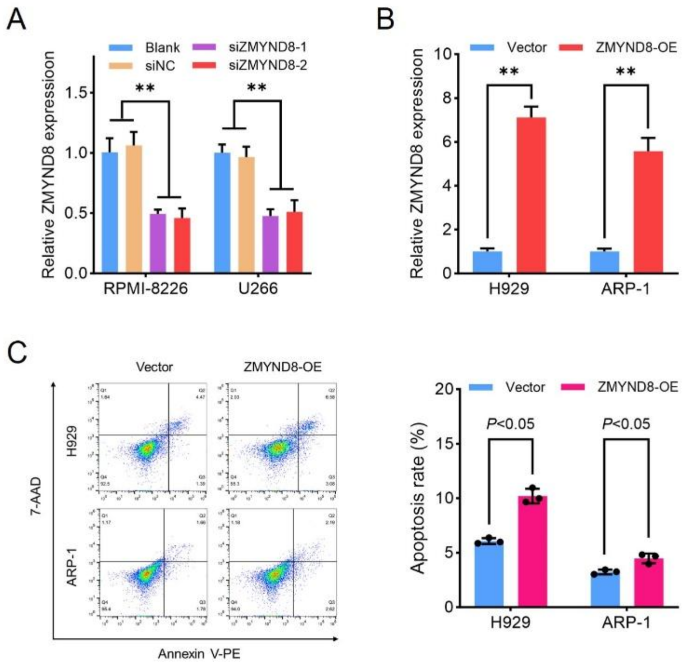

Figure S3. (A) Relative mRNA expression of ZMYND8 analyzed via qPCR in RPMI-8226 and U266 cells transfected with the negative control (siNC) or siZMYND8. Data in A are presented as mean ± SD, ** $p < 0.01$ by one-way ANOVA test. (B) Relative mRNA expression levels of ZMYND8 in H929 and ARP-1 cells stably transfected with vector or ZMYND8-overexpression (OE) plasmids. (C) Cell apoptosis analyzed by flow cytometry in H929 and ARP-1 cells stably transfected with vector or ZMYND8-OE plasmids. Data in B and C are presented as mean ± SD, ** $p < 0.01$ by Student's *t*-test.

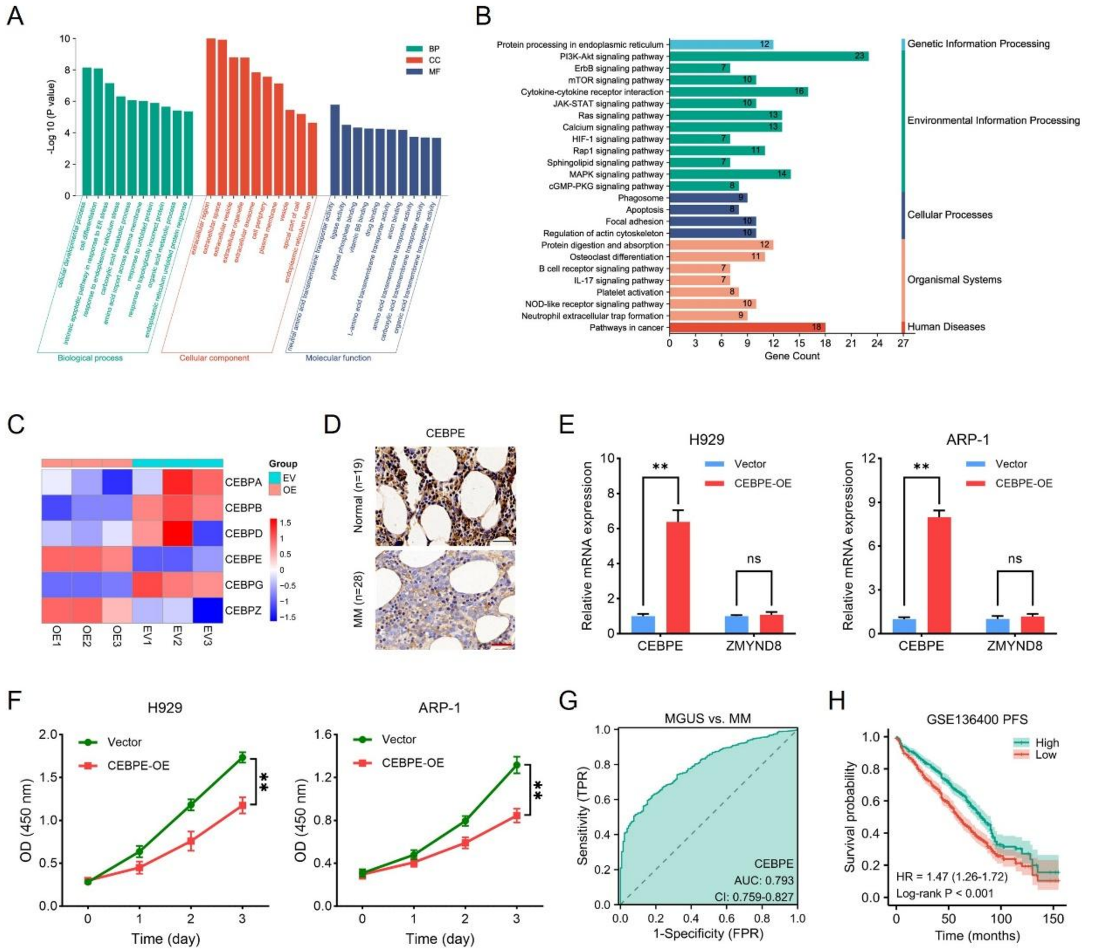

Figure S4. (A) GO functional enrichment analysis of differentially expressed genes in RNA-seq, including three modules: biological process (BP), cellular component (CC), and molecular function (MF). The top 10 most significant pathways for each component are presented. (B) KEGG pathway enrichment results of transcriptomic differentially expressed genes, which is divided into five categories, including genetic information processing, environmental information processing, cellular processes, organismal systems, and human diseases. (C) Heatmap showing the differential mRNA expression of CEBP family genes (CEBPA, CEBPB, CEBPD, CEBPE, CEBPG, and CEBPZ) between empty vector (EV) and ZMYND8-OE groups. (D) Representative IHC staining images of CEBPE protein in bone marrow specimens from heathy donors (n = 19) and MM patients (n = 28). (E) Relative mRNA levels of CEBPE and ZMYND8 analyzed via qPCR in vector control and CEBPE-OE groups of H929 and ARP-1 cells. Data in E are presented as mean ± SD, ** $p < 0.01$ by Student's t-test; ns, non-significant. (F) Cell proliferation assays using CCK-8 after CEBPE overexpression in H929 and ARP-1 cells. Data in F are presented as mean ± SD, ** $p < 0.01$ by two-way ANOVA test. (G) ROC curve analysis of CEBPE expression in the discrimination between MGUS and MM in the GSE136400 dataset. (H) Kaplan-Meier curves of PFS for patients with high and low CEBPE expression in the GSE136400 dataset.

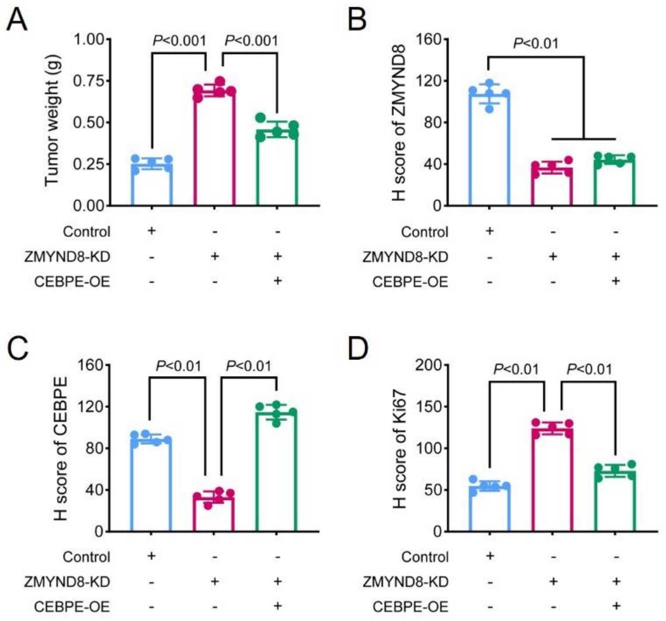

Figure S5. (A) Tumor weight of xenografts in the control, ZMYND8-knockdown (KD), and ZMYND8-KD + CEBPE-OE groups. (B–D) *H*-score analysis of ZMYND8 (B), CEBPE (C), and Ki67 (D) in tumor xenografts excised from mice in the indicated experimental groups. Data in A–D are presented as mean ± SD and analyzed by one-way ANOVA test ($n = 5$).

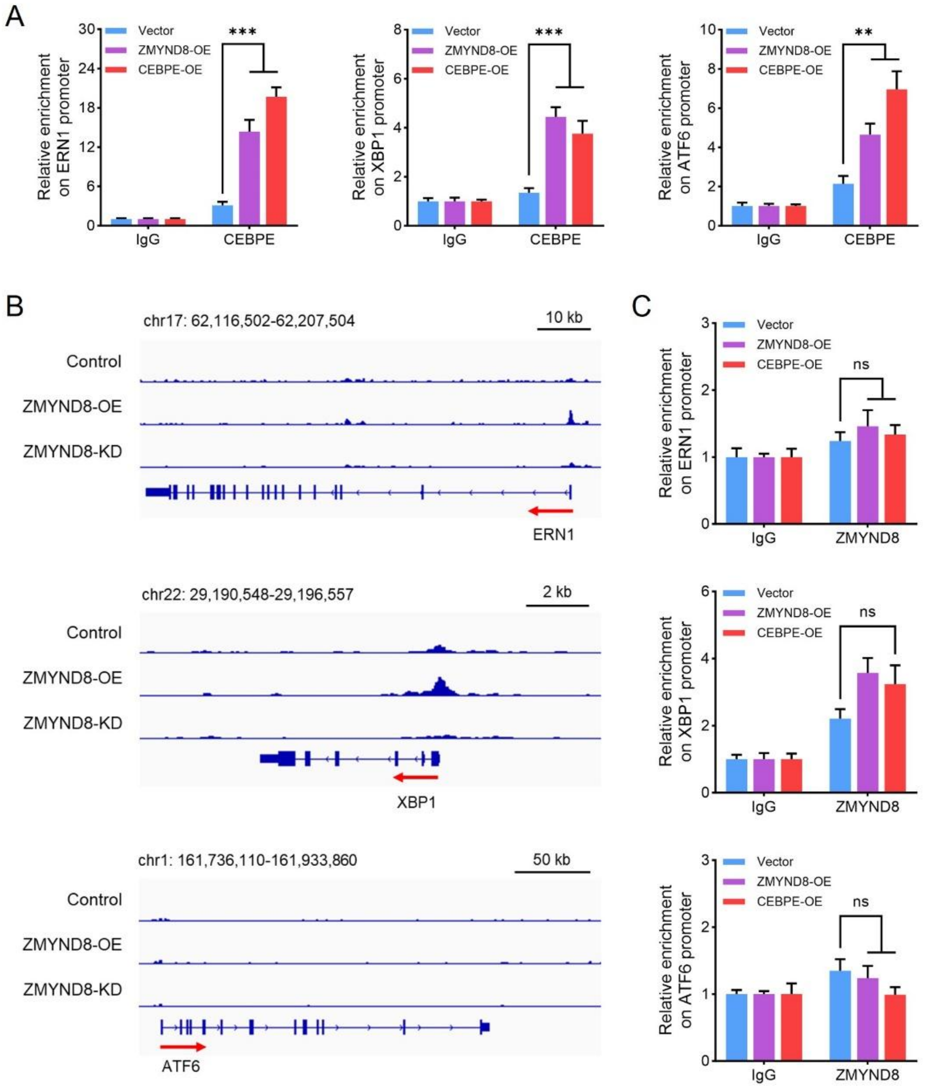

Figure S6. (A) ChIP–qPCR analysis of relative CEBPE enrichment within the promoter regions of ERN1 (left), XBP1 (middle), and ATF6 (right) in ARP-1 cells transfected with the vector control, ZMYND8-OE, or CEBPE-OE plasmid. (B) CUT&Tag sequencing visualizing the enrichment abundance of ZMYND8 at the loci of ERN1 (upper), XBP1 (middle), and ATF6 (lower) in the control, ZMYND8-OE, and ZMYND8-KD groups. (C) ChIP–qPCR analysis of the enrichment levels of ZMYND8 at the promoter regions of ERN1 (upper), XBP1 (middle), and ATF6 (lower) in the vector control, ZMYND8-OE, and CEBPE-OE groups. Fold enrichment was calculated relative to the IgG control. Data in A and C are presented as mean ± SD, ** $p < 0.01$, *** $p < 0.001$ by two-way ANOVA test; ns, non-significant.

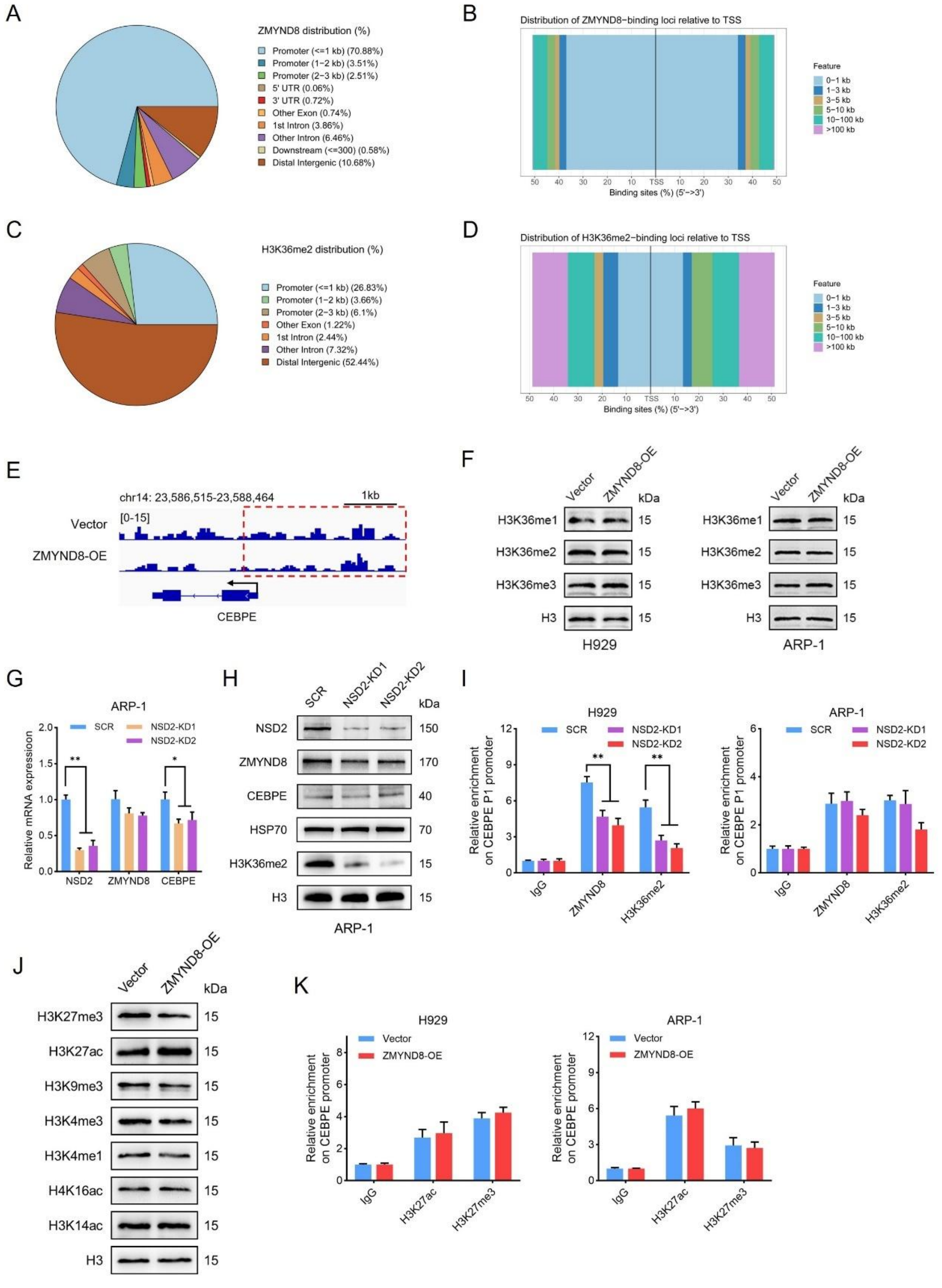

Figure S7. (A) Global distribution proportion of ZMYND8 peaks. (B) Distribution of ZMYND8 peaks relative to the transcriptional start site (TSS). (C) Global distribution proportion of H3K36me2 peaks. (D) Distribution of H3K36me2 peaks relative to the TSS. (E) Genomic tracks of H3K36me2 at the CEBPE gene loci in the ZMYND8-OE group compared with the vector control group in H929 cells. (F) Western blot analysis of H3K36me1, H3K36me2, and H3K36me3 levels after ZMYND8 overexpression in H929 and ARP-1 cells.

Histone H3 was used as reference control. (G) Relative mRNA expression of NSD2, ZMYND8, and CEBPE after NSD2 knockdown in ARP-1 cells. Data in G are presented as mean ± SD, * $p < 0.05$, ** $p < 0.01$ by one-way ANOVA test. (H) Western blot analysis of expression levels of NSD2, ZMYND8, CEBPE, and H3K36me2 following NSD2 knockdown in ARP-1 cells. (I) ChIP–qPCR analysis of ZMYND8 and H3K36me2 enrichment in the CEBPE promoter P1 region in H929 (left) and ARP-1 (right) cells. (J) Western blot analysis of the indicated histone modifications in the vector control and ZMYND8-OE H929 cells. Histone H3 was used as a loading control. (K) ChIP–qPCR analysis of H3K27ac and H3K27me3 enrichment at the CEBPE promoter in the vector control and ZMYND8-OE groups of H929 (left) and ARP-1 (right) cells. Data in I and K are presented as mean ± SD, ** $p < 0.01$ by two-way ANOVA test.

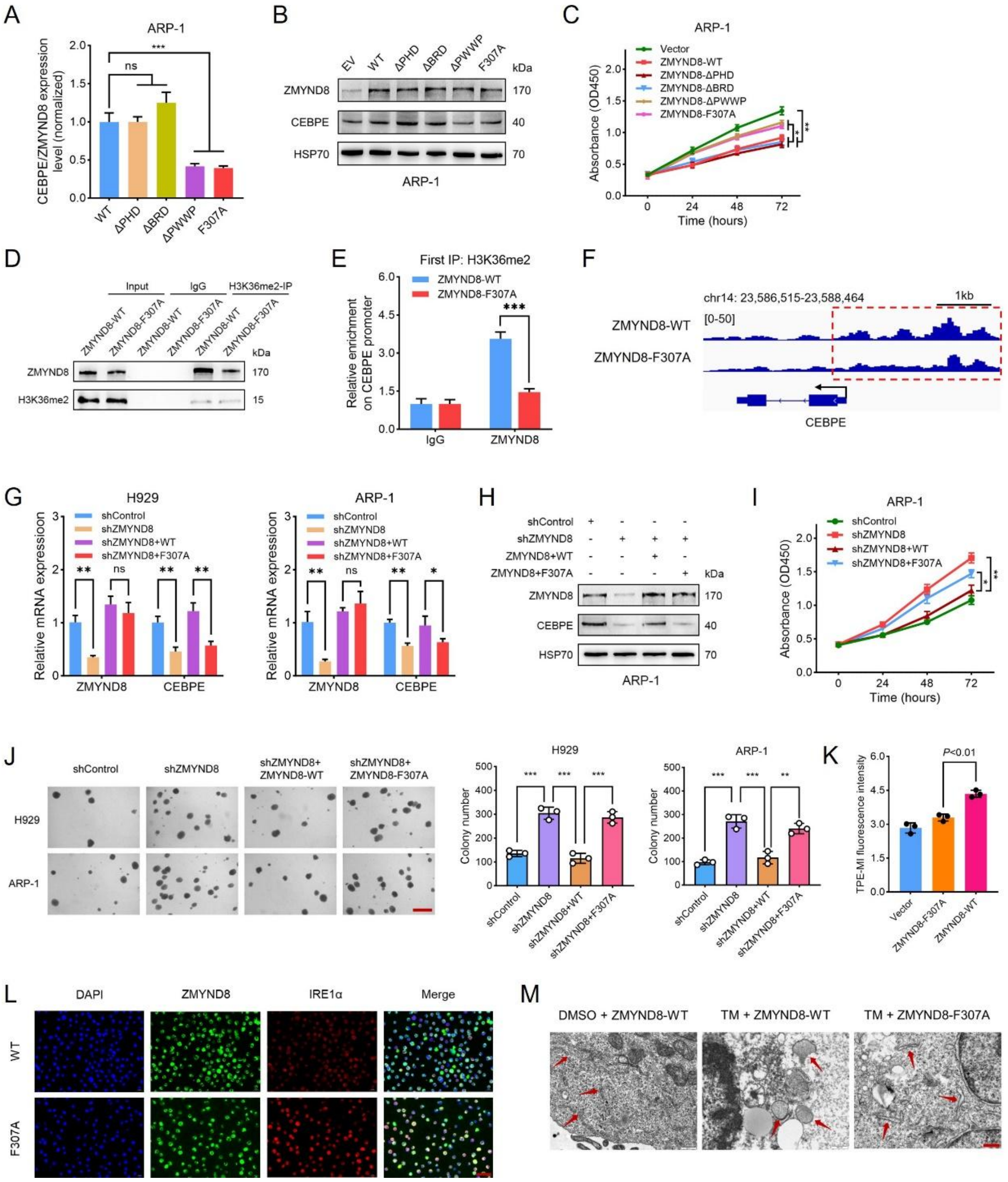

Figure S8. (A) Relative CEBPE/ZMYND8 mRNA level in ARP-1 cells transfected with ZMYND8 overexpression plasmids including ZMYDN8-WT, ΔPHD, ΔBRD, ΔPWWP, and F307A. Data in A are presented as mean ± SD, *** $p < 0.001$ by one-way ANOVA test; ns, non-significant. (B) Western blot analysis of ZMYND8 and CEBPE expression in ARP-1 cells transfected with the empty vector (EV), ZMYND8-WT, or ZMYND8 mutant plasmids. (C) CCK-8 assays in ARP-1 cells after transfection with ZMYND8 overexpression or vector plasmids. (D) Co-IP assay with lysates extracted from ZMYND8-KD ARP-1 cells transfected with the ZMYND8-WT or ZMYND8-F307A plasmid. Anti-H3K36me2 antibody was used for immunoprecipitation, and IgG served as a negative control. (E) ChIP–reChIP analysis of ZMYND8 enrichment level on the CEBPE promoter in ZMYND8-KD ARP-1 cells after

transfection with the ZMYND8-WT or ZMYND8-F307A plasmid. Anti-H3K36me2 antibody was used for first immunoprecipitation. Data in E are presented as mean ± SD, *** $p < 0.001$ by two-way ANOVA test. (F) Genomic tracks of ZMYND8 at the CEBPE gene loci in the ZMYND8-F307A overexpression group compared with the ZMYND8-WT overexpression group (same as the ZMYND8-OE group in Figure 3F) in H929 cells. (G) Relative mRNA levels of ZMYND8 and CEBPE in ZMYND8-KD H929 (left) and ARP-1 (right) cells transfected with the ZMYND8-WT or ZMYND8-F307A plasmid. Data in G are presented as mean ± SD, * $p < 0.05$, ** $p < 0.01$ by one-way ANOVA test; ns, non-significant. (H) Western blot analysis of ZMYND8 and CEBPE expression in ZMYND8-KD ARP-1 cells transfected with the ZMYND8-WT or ZMYND8-F307A plasmid. (I) CCK-8 assays in ZMYND8-KD ARP-1 cells after transfection with the ZMYND8-WT or ZMYND8-F307A plasmid. Data in C and I are presented as mean ± SD, * $p < 0.05$, ** $p < 0.01$ by two-way ANOVA test. (J) Methylcellulose clone formation assays in ZMYND8-KD H929 and ARP-1 cells after transfection with the ZMYND8-WT or ZMYND8-F307A plasmid. The scale bar represents 300 μm. Data in J are presented as mean ± SD, ** $p < 0.01$, *** $p < 0.001$ by one-way ANOVA test. (K) Median fluorescence intensity of tetraphenylethene maleimide (TPE-MI) measured by flow cytometry for H929 cells in the vector, ZMYND8-F307A, and ZMYND8-WT groups under tunicamycin (TM) treatment. Data in K are presented as mean ± SD and analyzed by one-way ANOVA test. (L) Immunofluorescence was performed for the detection of ZMYND8 and IRE1α in H929 cells transfected with the ZMYND8-WT or ZMYND8-F307A plasmid. The scale bar represents 50 μm. (M) Transmission electron microscopy (TEM) was used to observe the ultrastructure of endoplasmic reticulum of H929 cells in the DMSO + ZMYND8-WT, TM + ZMYND8-WT, and TM + ZMYND8-F307A groups. Red arrows indicate representative endoplasmic reticulum. The scale bar represents 0.5 μm.

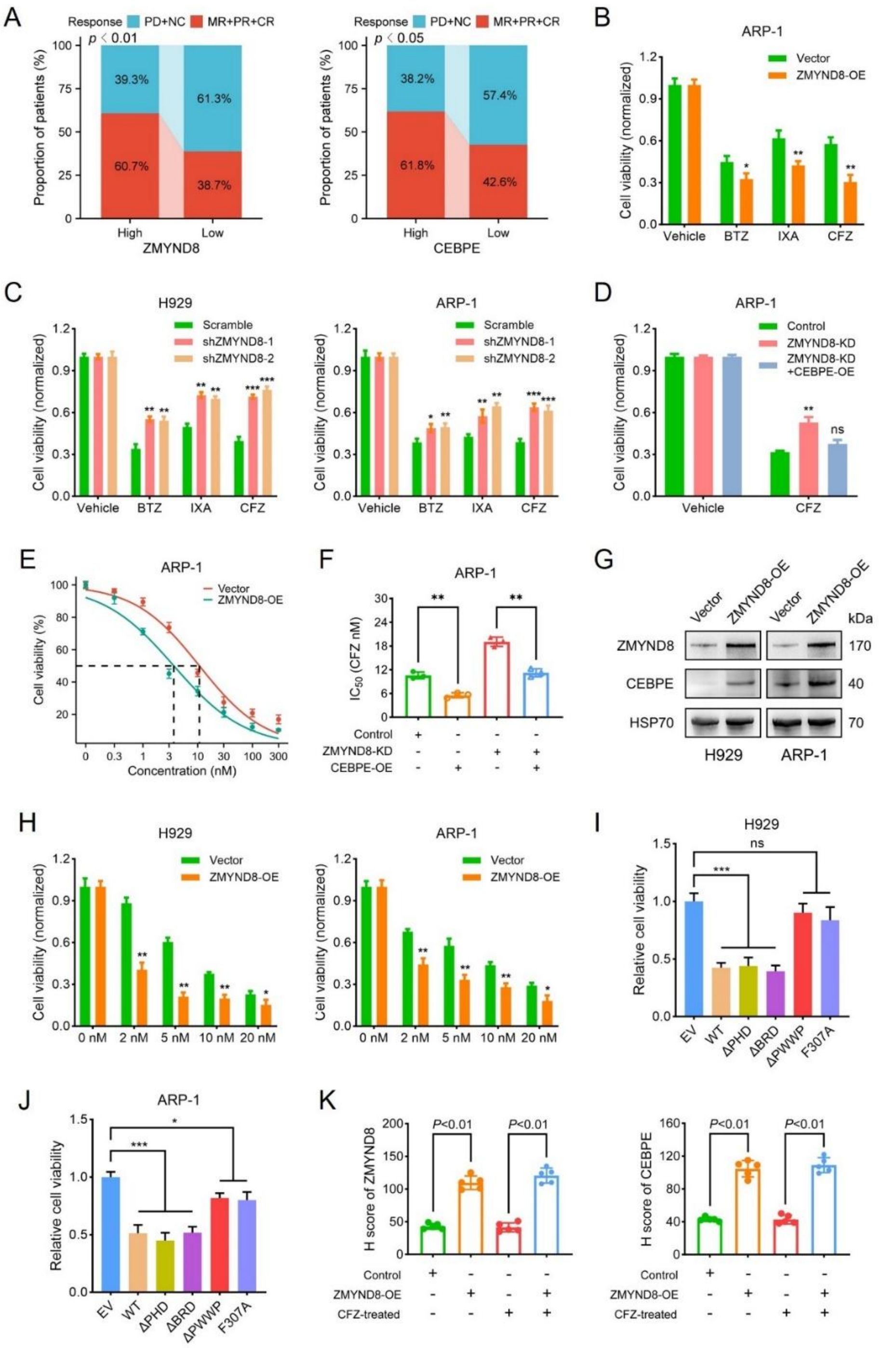

Figure S9. (A) Proportion of patients with different clinical response to PI therapy categorized by the expression levels of ZMYND8 (left) or CEBPE (right). Data in A were compared by Chi-squared test. (B) Cell viability was determined 36 h after treatment with DMSO (vehicle), 10 nM BTZ, 10 nM IXA, or 10 nM CFZ in ARP-1 cells transfected with vector or ZMYND8-OE plasmids. Data in B are presented as mean ± SD, * $p < 0.05$, ** $p < 0.01$ by Student's *t*-test. (C) Cell viability was detected 48 h after treatment with DMSO (vehicle), 10 nM BTZ, 10 nM IXA, or 10 nM CFZ in H929 (left) and ARP-1 (right) cells transfected with the scramble, shZMYND8-1, or shZMYND8-2 plasmids. (D) Cell viability was determined 48 h after

treatment with DMSO or 10 nM CFZ in ARP-1 cells treated with the negative control, ZMYND8-KD, or ZMYND8-KD + CEBPE-OE. Data in C and D are presented as mean ± SD, * $p < 0.05$, ** $p < 0.01$, *** $p < 0.001$ by one-way ANOVA test; ns, non-significant. (E) Comparison of the IC_{50} values of CFZ between vector control and ZMYND8-OE groups in ARP-1 cells. (F) IC_{50} values of CFZ in ARP-1 cells treated with the negative control, ZMYND8-KD, CEBPE-OE, or ZMYND8-KD + CEBPE-OE. Data in F are presented as mean ± SD, ** $p < 0.01$ by one-way ANOVA test. (G) Protein levels of CEBPE in the vector and ZMYND8-OE groups in H929 and ARP-1 cells under CFZ treatment (10 nM for 48 h). (H) Cell viability was determined 48 h after treatment with a concentration gradient of CFZ in H929 and ARP-1 cells transfected with vector or ZMYND8-OE plasmids. Data in H are presented as mean ± SD, * $p < 0.05$, ** $p < 0.01$ by Student's t-test. (I, J) Relative cell viability of H929 cells (I) and ARP-1 cells (J) transfected with empty vector (EV) or ZMYND8 overexpression plasmids (ZMYND8-WT, ΔPHD, ΔBRD, ΔPWWP, and F307A) after treatment with 10 nM CFZ. Data in I and J are presented as mean ± SD, * $p < 0.05$, *** $p < 0.001$ by one-way ANOVA test; ns, non-significant. (K) H-score analysis of ZMYND8 (left) and CEBPE (right) proteins in tumor xenografts excised from mice in each experimental group. Data in K are presented as mean ± SD and analyzed by one-way ANOVA test.

Table S1. The baseline characteristics of 102 MM patients and associations of ZMYND8 expression level with clinicopathological features

Characteristics	All patients N=102	ZMYND8^{high} N=51	ZMYND8^{low} N=51	*P* value
Age (years), mean (SD)	59.0 (8.8)	57.0 (7.4)	60.9 (9.6)	0.022
Sex, n (%)				1.000
Male	63 (61.8%)	32 (62.7%)	31 (60.8%)	
Female	39 (38.2%)	19 (37.3%)	20 (39.2%)	
Isotype, n (%)				0.613
IgG	49 (48.0%)	22 (43.1%)	27 (52.9%)	
IgA	27 (26.5%)	15 (29.4%)	12 (23.5%)	
FLC	22 (21.6%)	11 (21.6%)	11 (21.6%)	
Other	4 (3.9%)	3 (5.9%)	1 (2.0%)	
BMPC (%), median [IQR]	24.2 [10.1, 51.5]	19.0 [7.5, 40.2]	36.0 [14.0, 62.8]	0.009
ALB (g/dL), median [IQR]	3.4 [2.9, 3.9]	3.6 [3.0, 4.0]	3.1 [2.8, 3.7]	0.069
BMG (mg/L), median [IQR]	3.6 [2.8, 5.6]	3.4 [2.6, 4.5]	4.5 [3.0, 7.1]	0.018
HGB (g/dL), median [IQR]	9.2 [7.6, 11.1]	9.8 [8.0, 11.5]	8.6 [6.8, 10.7]	0.037
CREAT (mg/dL), median [IQR]	0.9 [0.7, 1.3]	0.9 [0.7, 1.3]	1.0 [0.8, 1.4]	0.237
LDH (U/L), median [IQR]	163.0 [143.2, 235.8]	151.0 [130.5, 206.5]	213.0 [152.5, 260.0]	0.002
High-risk CA, n (%)				0.014
Yes	39 (38.2%)	13 (25.5%)	26 (51.0%)	
No	63 (61.8%)	38 (74.5%)	25 (49.0%)	
Diabetes mellitus, n (%)				0.553
Yes	13 (12.7%)	8 (15.7%)	5 (9.8%)	
No	89 (87.3%)	43 (84.3%)	46 (90.2%)	
Hypertension, n (%)				0.506
Yes	28 (27.5%)	16 (31.4%)	12 (23.5%)	
No	74 (72.5%)	35 (68.6%)	39 (76.5%)	
D-S stage, n (%)				0.327
I-II	21 (20.6%)	13 (25.5%)	8 (15.7%)	
III	81 (79.4%)	38 (74.5%)	43 (84.3%)	
ISS stage, n (%)				0.009
I	29 (28.4%)	20 (39.2%)	9 (17.6%)	
II	45 (44.1%)	23 (45.1%)	22 (43.1%)	
III	28 (27.5%)	8 (15.7%)	20 (39.2%)	
R-ISS stage, n (%)				0.003
I	21 (20.6%)	17 (33.3%)	4 (7.8%)	
II	58 (56.9%)	27 (52.9%)	31 (60.8%)	
III	23 (22.5%)	7 (13.7%)	16 (31.4%)	
Treatment regimens, n (%)				0.795
PIs-based	84 (82.4%)	43 (84.3%)	41 (80.4%)	
Traditional drugs-based	18 (17.6%)	8 (15.7%)	10 (19.6%)	
ASCT receipt, n (%)				0.661

Yes	29 (28.4%)	16 (31.4%)	13 (25.5%)
No	73 (71.6%)	35 (68.6%)	38 (74.5%)

SD, standard deviation; IQR, interquartile range; FLC, free light chain; BMPC, bone marrow plasma cell; ALB, albumin; BMG, β2-microglobulin; HGB, hemoglobin; CREAT, creatinine; LDH, lactate dehydrogenase; CA, cytogenetic abnormality; D-S, Durie-Salmon; ISS, International Staging System; R-ISS, Revised-International Staging System; PIs, proteasome inhibitors; ASCT, autologous stem cell transplantation. *P* value is for comparison between ZMYND8 high and low expression groups.

Table S2. Univariate and multivariate Cox regression analysis for OS

**Characteristics**	**Univariate analysis**			**Multivariate analysis**		
	**HR**	**95% CI**	***P* value**	**HR**	**95% CI**	***P* value**
Age	1.042	1.005-1.080	0.025	0.992	0.950-1.036	0.723
Sex (female vs. male)	1.091	0.543-2.194	0.807			
BMPC	1.008	0.997-1.020	0.149			
ALB	0.594	0.378-0.934	0.024	0.732	0.431-1.242	0.247
BMG	1.070	0.979-1.168	0.134			
HGB	0.930	0.818-1.058	0.272			
CREAT	1.068	0.894-1.277	0.468			
LDH	1.006	1.002-1.009	0.001	1.004	1.001-1.007	0.023
Diabetes mellitus (no vs. yes)	1.057	0.317-3.517	0.928			
Hypertension (no vs. yes)	1.102	0.475-2.557	0.821			
High-risk CA (yes vs. no)	3.432	1.618-7.280	0.001	2.757	1.188-6.396	0.018
ZMYND8 expression (low vs. high)	3.640	1.504-8.810	0.004	2.770	1.109-6.920	0.029

OS, overall survival; BMPC, bone marrow plasma cell; ALB, albumin; BMG, β2-microglobulin; HGB, hemoglobin; CREAT, creatinine; LDH, lactate dehydrogenase; CA, cytogenetic abnormality.

Table S3. Univariate and multivariate Cox regression analysis for PFS

Characteristics	Univariate analysis			Multivariate analysis		
	HR	95% CI	*P* value	HR	95% CI	*P* value
Age	1.021	0.989-1.055	0.207			
Sex (female vs. male)	0.991	0.535-1.837	0.978			
BMPC	1.020	1.009-1.031	<0.001	1.019	1.006-1.032	0.005
ALB	0.529	0.328-0.854	0.009	1.123	0.674-1.871	0.655
BMG	1.066	0.992-1.145	0.081			
HGB	0.945	0.839-1.064	0.349			
CREAT	1.186	1.009-1.393	0.038	1.295	1.016-1.651	0.037
LDH	1.005	1.002-1.008	<0.001	1.004	1.001-1.007	0.010
Diabetes mellitus (no vs. yes)	1.137	0.446-2.901	0.788			
Hypertension (no vs. yes)	0.764	0.395-1.476	0.422			
High-risk CA (yes vs. no)	3.623	1.935-6.783	<0.001	1.929	0.947-3.931	0.070
ZMYND8 expression (low vs. high)	3.186	1.599-6.351	0.001	2.873	1.374-6.007	0.005

PFS, progression-free survival; BMPC, bone marrow plasma cell; ALB, albumin; BMG, β2-microglobulin; HGB, hemoglobin; CREAT, creatinine; LDH, lactate dehydrogenase; CA, cytogenetic abnormality.

Table S4. Primer sequences for siRNA, shRNA, and mutant construction of ZMYND8

Name	Direction	Sequence information (5'-3')
siZMYND8-1	N/A	GAACAAUGACAGAAAUAUA
siZMYND8-2	N/A	GAGUUGCAAAGCACAAUUA
Scramble (SCR)	Forward	CCGGCCTAAGGTTAAGTCGCCCTCGCTCGAGCG AGGGCGACTTAACCTTAGGTTTTTG
	Reverse	AATTCAAAAACCTAAGGTTAAGTCGCCCTCGCT CGAGCGAGGGCGACTTAACCTTAGG
shZMYND8-1	Forward	CCGGGCTGCAGAAATCCTTCAACTTCTCGAGAA GTTGAAGGATTTCTGCAGCTTTTTG
	Reverse	AATTCAAAAAGCTGCAGAAATCCTTCAACTTCT CGAGAAGTTGAAGGATTTCTGCAGC
shZMYND8-2	Forward	CCGGCCGGATTTCCTTGTCGGATATCTCGAGATA TCCGACAAGGAAATCCGGTTTTTG
	Reverse	AATTCAAAAACCGGATTTCCTTGTCGGATATCTC GAGATATCCGACAAGGAAATCCGG
ΔPHD	Forward	GGACGGAATACAGTAGCAGAATGCATCGAGACC
	Reverse	TGCTACTGTATTCCGTCCATCCTGCTCCTTATT
ΔBRD	Forward	GTTTGCCATTCAGAAACAAATAGCGAAAGTAGT CATCAAAATC
	Reverse	TGTTTCTGAATGGCAAACTTGAGCAGGTAGGAT
ΔPWWP	Forward	GCCTTGTAGCAATATTCCTTTTTCTGTGAAAAAG ACTAAGA
	Reverse	GGAATATTGCTACAAGGCTCACAAAACCAGTTA
F307A	Forward	CGAGCCTTTGGACAACATGACAGGGCCTGGGTT
	Reverse	ATGTTGTCCAAAGGCTCGGGCATCGACCTGCCC

Table S5. Primer sequences for qRT-PCR analyses

Gene symbol	Forward primer (5'-3')	Reverse primer (5'-3')
GAPDH	CGGAGTCAACGGATTTGGTC	TTCCCGTTCTCAGCCTTGAC
ZMYND8	TGCATCCACAGAGTCCCAAAT	ATGGAGGAAGGCTGCATTCAA
CEBPE	TTGCCTACCCTCCACATACCT	CTCCTCCTTCACCGCCACAGC
CCT8L2	CCTGCCCATCCAAATGCAC	GCTGGCCTACTTGCTTTTCTA
LGR6	AGCCCTGTGAGTACCTCTTTG	CAGCACCAGTCCATTGCAGA
AQP1	CTGGGCATCGAGATCATCGG	ATCCCACAGCCAGTGTAGTCA
RHBDL3	CGCCATGTGGCCTATGAGAC	TGACACCCCATTGTAGAGGAA
SLC4A1	CCTATACGCTTCCTCTTTGTGTT	CCATGTAGGCATCTATGCGGA
CSPG4	AGGACGAAGGAACCCTAGAGT	CACAGGCACACTGTTGTGGA
CCDC146	TGCCATAGTGCCCACAATTAAC	TTGGCTTTTAACGCTGCCATT
KCNT2	GGTACAGGTTTCGAGATTTGCT	ACAGGCGTATCCTTAGACTTGA
BFSP1	TGCCCATGAGTGTTATGACGA	GCCTTCAATCTCGATGATACGAT
MAFA	GAGCGGCTACCAGCATCAC	CTCTGGAGTTGGCACTTCTCG
SH3TC2	TCAGCAAGGTTGGTCAGTATCC	CCAATATGCAGTTGAGCCAGT
ACTL10	ATCTACGCGGGTCACTCGT	CCCGTTACCCACGCTGAAA
CNIH2	CTGGCACATCATAGCCTTTGA	AAGAGGCCGTGGATGGAGTAT
LYPD3	GGTCGTGAGCTGCTACAAC	GGTCAGAGTTACAGCGGGAC
CHAC1	GGCTACAGCCGCCGTTTCT	CACTGCCTCTCGCACATTC
TRIB3	GATGCCCTTAGCCCCAACCC	GAACCACTTCTCTGTCTCCC
ERN1	AGAGAAGCAGCAGACTTTGTC	GTTTTGGTGTCGTACATGGTGA
XBP1	TGGTTGCTGAAGAGGAGGCG	TGGGGAGATGTTCTGGAGGG
CEBPB	AACTCTCTGCTTCTCCCTCTG	AAGCCCGTAGGAACATCTTT
HERPUD1	TTATTCTGGGAAGCTGTTGT	GTTGATTTCTGGCATTTTTG
BBC3	GACCTCAACGCACAGTACGAG	AGGAGTCCCATGATGAGATTGT
DDIT3	TTGACCCTGCTTCTCTGGCTT	TCCGTTTCCTGGTTCTCCCTT
ATF6	CCCGTATTCTTCAGGGTGCT	ACTCCCTGAGTTCCTGCTGA
ATP2A3	AAGTGCCTGCTGACCTCCG	ACCCACCGCTTTGCCCGAT
ATF4	GTTGGTGGGGGACTTGATGT	TTAGCCTTGTCGCTGGAGAA
TXNDC12	TGGCAAGGTGCATCCTGAAAT	TGCTCGGCACTGACATAAAAA
ERP29	GGGGCAGTTAAGGTTGGAGC	TCTCCTTCACACTTGAGAGGTT
ATF3	CGGAGCCTGGAGCAAAATGA	GGATGGCAAACCTCAGCTCT
STC2	ACAGGTTCGGCTGCATAAGC	GAGGTCCACGTAGGGTTCG
NSD2	CGCAACCCATCAGAGTGTTCT	ACTGCCGAGGATTTCTGGTG

Table S6. Primer sequences for ChIP assays

Fragments	Direction	Sequence information (5'-3')
CEBPE promoter		
P1	Forward	TCAAAGTAGGGGAGGGAACA
	Reverse	CTGGAAGGAGGAAGAGGAAA
P2	Forward	GTAGGGAGGATGTAAGGAA
	Reverse	AGAGGGACCAGAAGTTTTT
P3	Forward	AAGGAAAGACAGCAAACGAG
	Reverse	CGGAGTAGACAGCCATAATC
P4	Forward	AACCCTGGCTTGGTCCCT
	Reverse	GCTCTGCTTCCTTTTCCT
P5	Forward	CGATCTCTTTGCCGTGAA
	Reverse	GGGCCGAAGGTATGTGGA
P6	Forward	CTCAAGGTAAGAGCAGGC
	Reverse	CACAGAGAAGGAAAGGGA
ERN1 promoter	Forward	CCCCCTCTGGTGCTTTTTCTC
	Reverse	GCATTCCCCTTTGTGTTTCAT
XBP1 promoter	Forward	CCGCAGAGGAGGAAGCAATAA
	Reverse	AGTCCGAGAAACCCCCGTAAA
ATF6 promoter	Forward	TATCTTTTTCTGGGCTTGCGT
	Reverse	GAACCCTCCAAACTGCTCGTG

Table S7. Information of antibodies used in the current study

Name	Vendor	Catalog number
Rabbit polyclonal anti-ZMYND8	Proteintech	11633-1-AP
Rabbit polyclonal anti-CEBPE	Proteintech	14271-1-AP
Rabbit polyclonal anti-XBP1	Abcam	ab37152
Rabbit polyclonal anti-ATF6	CST	D4Z8V
Rabbit polyclonal anti-IRE1α	CST	14C10
Rabbit polyclonal anti-NSD2	ABclonal	A7938
Rabbit polyclonal anti-GAPDH	Proteintech	10494-1-AP
Rabbit polyclonal anti-HSP70	ABclonal	A12948
Rabbit polyclonal anti-H3	ABclonal	A2348
Rabbit polyclonal anti-H3K36me1	Abcam	ab9048
Rabbit polyclonal anti-H3K36me2	Abcam	ab9049
Rabbit polyclonal anti-H3K36me3	Abcam	ab9050
Rabbit polyclonal anti-KI67	Proteintech	27309-1-AP
HRP Goat Anti-Rabbit IgG (H+L)	ABclonal	AS014
Rabbit polyclonal anti-H3K27me3	Proteintech	39156
Rabbit polyclonal anti-H3K27ac	Proteintech	39135
Rabbit polyclonal anti-H3K9me3	Proteintech	39161
Rabbit polyclonal anti-H3K4me1	ABclonal	A2355
Rabbit polyclonal anti-H3K4me3	ABclonal	A2357
Rabbit polyclonal anti-H3K14ac	ABclonal	A7254
Rabbit polyclonal anti-H4K16ac	ABclonal	A5280
